# Gas phase synthesis of the C40 nano bowl C_40_H_10_

**DOI:** 10.1038/s41467-023-37058-y

**Published:** 2023-03-18

**Authors:** Lotefa B. Tuli, Shane J. Goettl, Andrew M. Turner, A. Hasan Howlader, Patrick Hemberger, Stanislaw F. Wnuk, Tianjian Guo, Alexander M. Mebel, Ralf I. Kaiser

**Affiliations:** 1 Department of Chemistry and Biochemistry, Florida International University, Miami, FL 33199 USA; 2 Department of Chemistry, University of Hawai’i at Mānoa, Honolulu, HI 96822 USA; 3 Paul Scherrer Insitute, CH-5232, Villigen PSI, Villigen, Switzerland; 4 School of Pharmaceutical Science and Technology, Tianjin University, 92 Weijin Road, Tianjin, 370001 PR China; 5 Present Address: Department of Chemistry, Johns Hopkins University, Baltimore, MD 21218 USA

**Keywords:** Reaction kinetics and dynamics, Reaction mechanisms, Laboratory astrophysics

## Abstract

Nanobowls represent vital molecular building blocks of end-capped nanotubes and fullerenes detected in combustion systems and in deep space such as toward the planetary nebula TC-1, but their fundamental formation mechanisms have remained elusive. By merging molecular beam experiments with electronic structure calculations, we reveal a complex chain of reactions initiated through the gas-phase preparation of benzocorannulene (C_24_H_12_) via ring annulation of the corannulenyl radical (C_20_H_9_^•^) by vinylacetylene (C_4_H_4_) as identified isomer-selectively in situ via photoionization efficiency curves and photoion mass-selected threshold photoelectron spectra. In silico studies provided compelling evidence that the benzannulation mechanism can be expanded to pentabenzocorannulene (C_40_H_20_) followed by successive cyclodehydrogenation to the C40 nanobowl (C_40_H_10_) – a fundamental building block of buckminsterfullerene (C_60_). This high-temperature pathway opens up isomer-selective routes to nanobowls via resonantly stabilized free-radical intermediates and ring annulation in circumstellar envelopes of carbon stars and planetary nebulae as their descendants eventually altering our insights of the complex chemistry of carbon in our Galaxy.

## Abstract (web summary)

Nanobowls represent building blocks of fullerenes and nanotubes as detected in combustion systems and deep space, but their formation mechanisms in these environments have remained elusive. Here, the authors explore the gas-phase formation of benzocorannulene and beyond to the C_40_ nanobowl.

## Introduction

**Fig. 1:**
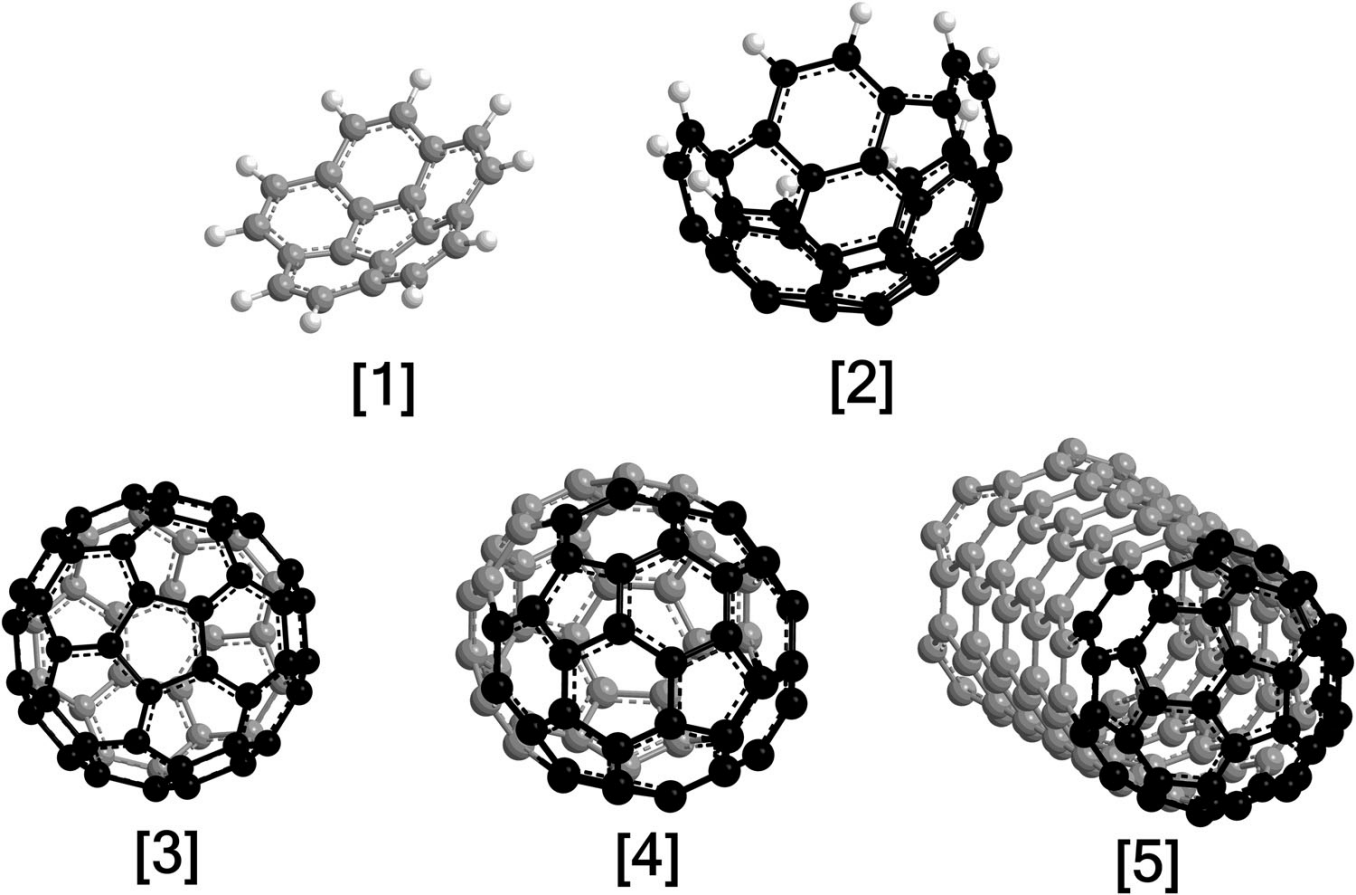
Molecular structures of 3-dimensional carbonaceous nanostructures. The smallest nanobowl, corannulene [1], as well as the C40 nanobowl [2] emphasized as a molecular building block of Buckminsterfullerene (C_60_) [3], rugbyballene (C_70_) [4], and end-capped (5,5) armchair nanotubes [5]. The C40 nanobowl carbons are black, the remaining carbons are gray, and the hydrogens are white.

The discovery of the fullerenes buckminsterfullerene (C_60_) and rugbyballene (C_70_)—allotropes of carbon consisting of carbon atoms connected by single and double bonds forming closed structures with five- and six-membered rings—nearly 40 years ago by Kroto, Heath, O’Brien, Smalley, and Curl^1^ has paved the path to remarkable scientific breakthroughs in material sciences, medicinal chemistry, physical chemistry, and organic chemistry^2^.This is due to the role of fullerenes as light-activated antimicrobial agents^3^, contrast agents^4^, molecular electronics^5^, lubricants^6^, and surface coatings^7^. In the post-fullerene era, edge-hydrogenated fullerene clusters attracted particular attention as molecular building blocks to form fullerenes and nanotubes from the bottom up. Here, the beauty of such often highly symmetric molecules triggered extensive interest from synthetic organic chemists who attempted to isolate the clusters by slicing the geodesic domes of fullerenes and saturating the dangling bonds of edge carbon atoms by hydrogen atoms^8–10^. Fullerene fragments, which belong to an extended family of curved (three-dimensional) polycyclic aromatic hydrocarbons (PAH), with the prototype species being the smallest bowl-shaped corannulene (C_20_H_10_) molecule (Fig. 1) are of special interest^11,12^. When arranged symmetrically in the condensed phase, such nanobowls afford an array of materials supported in changeable complex environments such as intermolecular charge transports^12^, which is rather distinct from the conventional mechanism based upon a tight overlap of π molecular orbitals in stacked carbonaceous materials.

Corannulene (C_20_H_10_), a stem circulene with a central polygon surrounded by six-membered aromatic rings, has been identified as the smallest bowl-shaped fragment of buckminsterfullene (C_60_), which also possesses the C_5_ rotational axis^8,13–16^. However, the synthesis of more complex buckybowls such as C40 (C_40_H_10_) and C50 (C_50_H_10_) nanobowls^12^ along with C39 (C_39_H_12_) and C46 (C_46_H_12_) nanobaskets^17^ has remained a fundamental synthetic challenge. Whereas the formation and isolation of these buckybowls are interesting by themselves from the synthetic chemistry viewpoint, the ultimate goal is to exploit these nanostructures as transitional templates to direct the synthesis of the closed icosahedral C_60_–fullerene structure and to employ them as end-caps of nanotubes^11,18,19^. Over the last decades, remarkable preparative organic synthetic routes have been reported to prepare mono- to pentabenzocorannulenes (C_40_H_20_) via addition of six-membered benzene rings, or benzannulation^20,21^, to the edge of corannulene^10,11,18,22,23^ but these pathways could not rationalize the synthetic routes to naturally prepared fullerenes detected in combustion flames^24^, in meteorites such as Allende and Murchison^25^, and in the planetary nebula TC-1^26^. Consequently, hitherto elusive high-temperature chemical routes for the synthesis of fullerenes along with their nanobowls must exist either from the bottom-up^27–29^ or from top-down^29–35^.

**Fig. 2:**
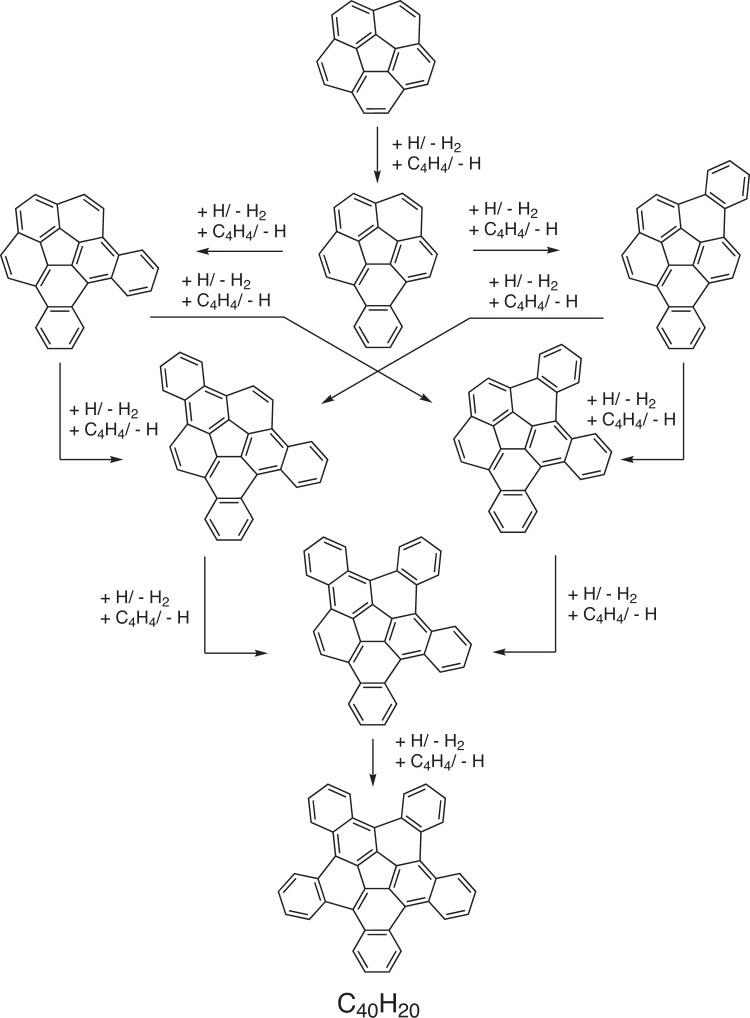
Pathways for the corannulenyl–vinylacetylene reaction. Schematic representation of reaction pathways to pentabenzocoranulene (C_40_H_20_).

Here, by combining molecular beam experiments with electronic structure calculations, we report on a complex chain of reactions initiated through the gas-phase preparation of benzocorannulene (C_24_H_12_) via ring annulation of the corannulenyl radical (C_20_H_9_^•^) by vinylacetylene (C_4_H_4_) as identified isomer-selectively in situ via fragment-free photoionization extracting photoionization efficiency curves (PIE) and photoion mass-selected threshold photoelectron spectra (ms-TPES). Exploiting benzocorannulene (C_24_H_12_) as a benchmark, we further expose in silico that the benzannulation mechanism can be expanded up to pentabenzocorannulene (C_40_H_20_) (Fig. 2), which encompasses two-thirds of the carbon content of C_60_, followed by successive cyclodehydrogenation to the C40 nanobowl (C_40_H_10_)^36^. In turn, the latter exhibits a deep bowl geometry and has curvature similar to that of buckminsterfullerene. The exploitation of linearly scaling coupled cluster method for electronic structure calculations of molecules as large as C_40_H_20_ opens a previously unavailable avenue for accurate exploration of potential energy diagrams of complex molecules with chemical accuracy allowing to obtain detailed information on energies of metastable states/products, barriers, and chemical pathways complementary to experimental data. This high-temperature route opens up a facile, isomer-selective bottom-up pathway to nanobowls via resonantly stabilized free-radical intermediates and ring annulation in combustion flames and in planetary nebulae, which may act as precursors to buckminsterfullerene (C_60_) thus changing our conception of the formation of complex carbon nanostructures in our Galaxy.

## Results

### Mass spectra

**Fig. 3:**
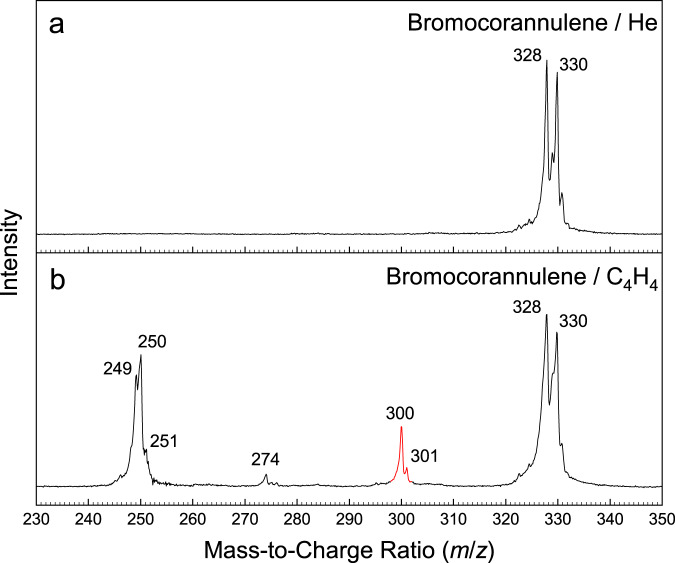
Photoionization mass spectra. Comparison of mass spectra taken at a photon energy of 9.00 eV. **a** Bromocorannulene (C_20_H_9_Br) – helium (He) system; **b** bromocorannulene (C_20_H_9_Br) – vinylacetylene (C_4_H_4_) system at a reactor temperature of 1200 ± 100 K. The mass peaks of the newly formed C_24_H_12_ (*m*/*z* = 300) species along with the ^13^C-substituted counterparts (*m*/*z* = 301) are highlighted in red.

A representative mass spectrum recorded at a photon energy of 9.00 eV for the reaction of corannulenyl radicals (C_20_H_9_^•^) with vinylacetylene (C_4_H_4_) is shown in Fig. 3b, while the mass spectrum for the reference experiment of bromocorannulene (C_20_H_9_Br) seeded in helium (He) without vinylacetylene is shown in Fig. 3a. In both systems, prominent ion counts were observed at *m*/*z* = 328 (C_20_H_9_^79^Br^+^) and 330 (C_20_H_9_^81^Br^+^). These signals correspond to the ^79^Br and ^81^Br isotopes of the bromocorannulene precursor, respectively. Under pyrolysis conditions in the presence of vinylacetylene (Fig. 3b), additional peaks arose at *m*/*z* = 249, 250, 251, 274, 300, and 301. The former two can be assigned to the molecular formulas C_20_H_9_^•+^ and C_20_H_10_^+^ indicating the corannulenyl radical formed via pyrolysis of bromocorannulene and corannulene generated through hydrogen abstraction and/or addition of atomic hydrogen, respectively. Signal at *m*/*z* = 251 can be connected to ^13^C-substituted corannulene, whereas ion counts at *m*/*z* = 274 likely originate from the reaction of corannulenyl (C_20_H_9_^•^; 249 amu) with acetylene (C_2_H_2_; 26 amu; impurity from the vinylacetylene cylinder) resulting in C_22_H_10_ isomer(s) attributed to 2-ethynylcorannulene and/or cyclopenta[*c,d*]corannulene. Finally, these data provide evidence that peaks at *m*/*z* = 300 and 301 are associated with the corannulenyl (C_20_H_9_^•^; 249 amu)–vinylacetylene (C_4_H_4_; 52 amu) reaction resulting in the formation of C_24_H_12_ isomer(s) and their ^13^C-substituted counterpart(s).

### PIE curves

**Fig. 4:**
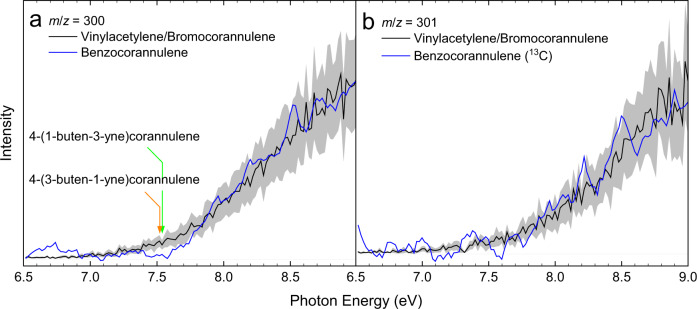
Photoionization efficiency (PIE) curves. PIE curves relevant to the formation of benzocorannulene. **a** *m*/*z* = 300 (C_24_H_12_) and **b** *m*/*z* = 301 (^13^CC_23_H_12_). Black: experimentally derived PIE curve; blue: benzocorannulene PIE curve; green: calculated ionization onset of *trans*−4-(1-buten-3-yne)corannulene; orange: calculated ionization onset of *cis*−4-(3-buten-1-yne)corannulene. The overall error bars (gray area) consist of two parts: 1 σ error of the PIE curve averaged over the individual scans and ±10% based on the accuracy of the photodiode.

**Fig. 5:**
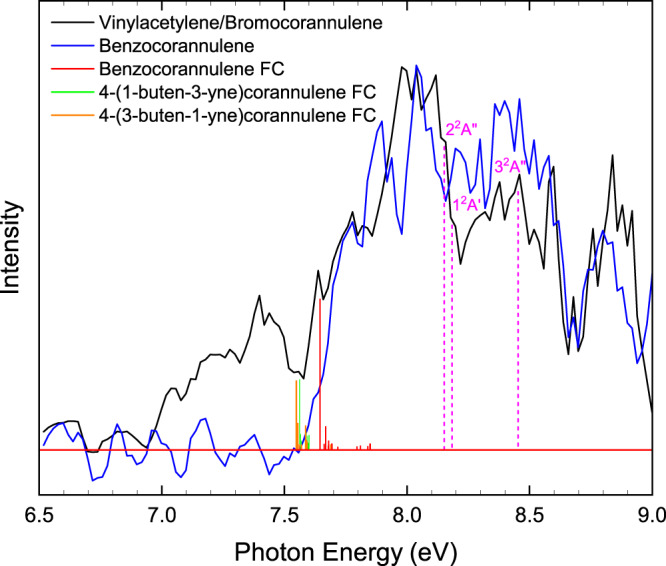
Mass-selected threshold photoelectron (ms-TPE) spectra. Photoion ms-TPE spectra relevant to the formation of benzocorannulene at *m*/*z* = 300 (C_24_H_12_). Black: experimentally derived ms-TPE spectrum; blue: benzocorannulene ms-TPE spectrum; red: Franck-Condon (FC) stick spectrum for benzocorannulene; green: FC spectrum for *trans*−4-(1-buten-3-yne)corannulene; orange: FC spectrum for *cis*−4-(3-buten-1-yne)corannulene; magenta: calculated ionization energies of 8.152, 8.184, and 8.453 eV corresponding to the 2^2^A′′, 1^2^A′, and 3^2^A′′ electronic excited states of the benzocorannulene cation.

Having assigned a hydrocarbon molecule(s) with the molecular formula C_24_H_12_ formed from the reaction of the corannulenyl radical with vinylacetylene, the structural isomer(s) must now be identified. This is accomplished by thoroughly analyzing the corresponding PIE curve and ms-TPES of *m*/*z* = 300 (C_24_H_12_^+^), which are presented in Fig. 4a and Fig. 5 along with reference spectra. Let us focus on the PIE curve first. The experimentally derived PIE curve at *m*/*z* = 300 (black) reproduces the reference PIE curve of the benzocorannulene (blue) sample above 7.70 eV very well (Fig. 4a). However, the ionization onset for the experimental curve of 7.45 ± 0.05 eV (*m*/*z* = 300) and 7.40 ± 0.05 eV (*m*/*z* = 301) does not match the onset of 7.70 ± 0.05 eV for the benzocorannulene curve. This may indicate that additional species beyond benzocorannulene are present in the molecular beam. Alternative isomers 4-(1-buten-3-yne)corannulene and 4-(3-buten-1-yne)corannulene have calculated adiabatic ionization energies of 7.54 ± 0.05 eV and 7.53 ± 0.05 eV, respectively (Fig. 4a). The onset for these isomers lies below that of benzocorannulene (7.70 eV); therefore, 4-(1-buten-3-yne)corannulene and 4-(3-buten-1-yne)corannulene may contribute to signal intensity of the experimental PIE curve below 7.7 eV to about 7.5 eV. The remaining intensity of the experimental PIE curve below 7.5 eV exhibits a gradual rise from zero indicative of hot bands^37^. Consequently, signal from about 7.4 to 7.7 eV arises from hot bands and/or from the 4-(1-buten-3-yne) corannulene and 4-(3-buten-1-yne)corannulene isomers, while a comparison of the corannulenyl–vinylacetylene and benzocorannulene PIE curves indicates that up to 99% of the total ion counts of the corannulenyl–vinylacetylene curve at photon energies beyond 7.7 eV can be attributed to benzocorannulene. It is important to note that the PIE curve taken at *m*/*z* = 301 (Fig. 4b) is essentially superimposable on the *m*/*z* = 300 (Fig. 4a) curve after scaling (Supplementary Fig. 2). Consequently, the data at *m*/*z* = 301 can be attributed to ^13^C-substituted isomers (^13^CC_23_H_12_) of benzocorannulene at an abundance of ~26% to account for the 1.1% natural abundance of ^13^C. Therefore, our results on the PIE curves reveal the formation of benzocorannulene through the reaction of the corannulenyl radical with vinylacetylene.

### ms-TPE spectra

In addition, we validated our assignment by exploiting the ms-TPES data (Fig. 5). The ms-TPE spectra for the corannulenyl–vinylacetylene system (black) and benzocorannulene (blue) reference show clear structure and, like the PIE curves, expose an excellent overlap from about 7.7 eV to 9.0 eV; this experimental evidence reinforces our conclusion that the major product of the corannulenyl–vinylacetylene reaction is indeed benzocorannulene. Besides this experimental evidence, we provided further computational evidence on the formation of benzocorannulene. The calculated Franck-Condon (FC) factors for benzocorannulene (red), 4-(1-buten-3-yne)corannulene (green), and 4-(3-buten-1-yne)corannulene (orange) are shown as stick spectra in Fig. 5, while calculated ionization energies of 8.15, 8.18, and 8.45 eV for the 2^2^Aʺ, 1^2^Aʹ, and 3^2^Aʺ excited electronic states of the benzocorannulene cation are denoted by dashed vertical lines (magenta). Especially the 2^2^Aʺ and 1^2^Aʹ states are responsible for the broad band centered at ~8 eV in both the reference and experimental ms-TPE spectra. The origin transition of the benzocorannulene FC spectrum at 7.65 ± 0.10 matches the ionization onset of the benzocorannulene ms-TPE and PIE spectra. The bands between 8.0 and 8.5 eV of the ms-TPES are comprised of up to three excited states, which may prevent the visibility of the true ionization onset. All states have vibrational transitions, which may overlap leading to the broad features. The 4-(1-buten-3-yne)corannulene and 4-(3-buten-1-yne)corannulene FC spectra show intense transitions at 7.56 ± 0.05 and 7.55 ± 0.05, respectively, indicating that these isomers may account for some of the intensity of the experimental spectrum below the onset of benzocorannulene, while the signal below 7.5 eV is due to hot band transitions. Overall, combining the PIE curves and ms-TPE spectra analyses provides compelling evidence for the formation of at least benzocorannulene from the reaction of corannulenyl radicals with vinylacetylene. It shall be highlighted that the combined extraction of PIE and ms-TPES data represents an authenticated approach to identify complex organic molecules as demonstrated at state-of-the-art VUV beamlines at Soleil (France)^38–40^, the Swiss Light Source (Switzerland)^41–43^, and the Advanced Light Source (US)^44,45^.

## Discussion

We are exploiting now linearly scaling coupled cluster electronic structure calculations to explore the complex reactions leading ultimately to the gas-phase formation of the C40 nanobowl (C_40_H_10_). This is accomplished in three steps. First, having identified benzocorannulene (C_24_H_12_), we unravel the mechanism of the gas-phase formation of benzocorannulene. This reaction sequence serves as a prototype to benchmark the experimental findings with linearly scaling coupled cluster methods. Second, these computations are expanded to pentabenzocoranulene (C_40_H_20_) via successive benzannulations. Finally, five successive ring-closures are explored computationally to eventually prepare the C40 nanobowl (C_40_H_10_).

**Fig. 6:**
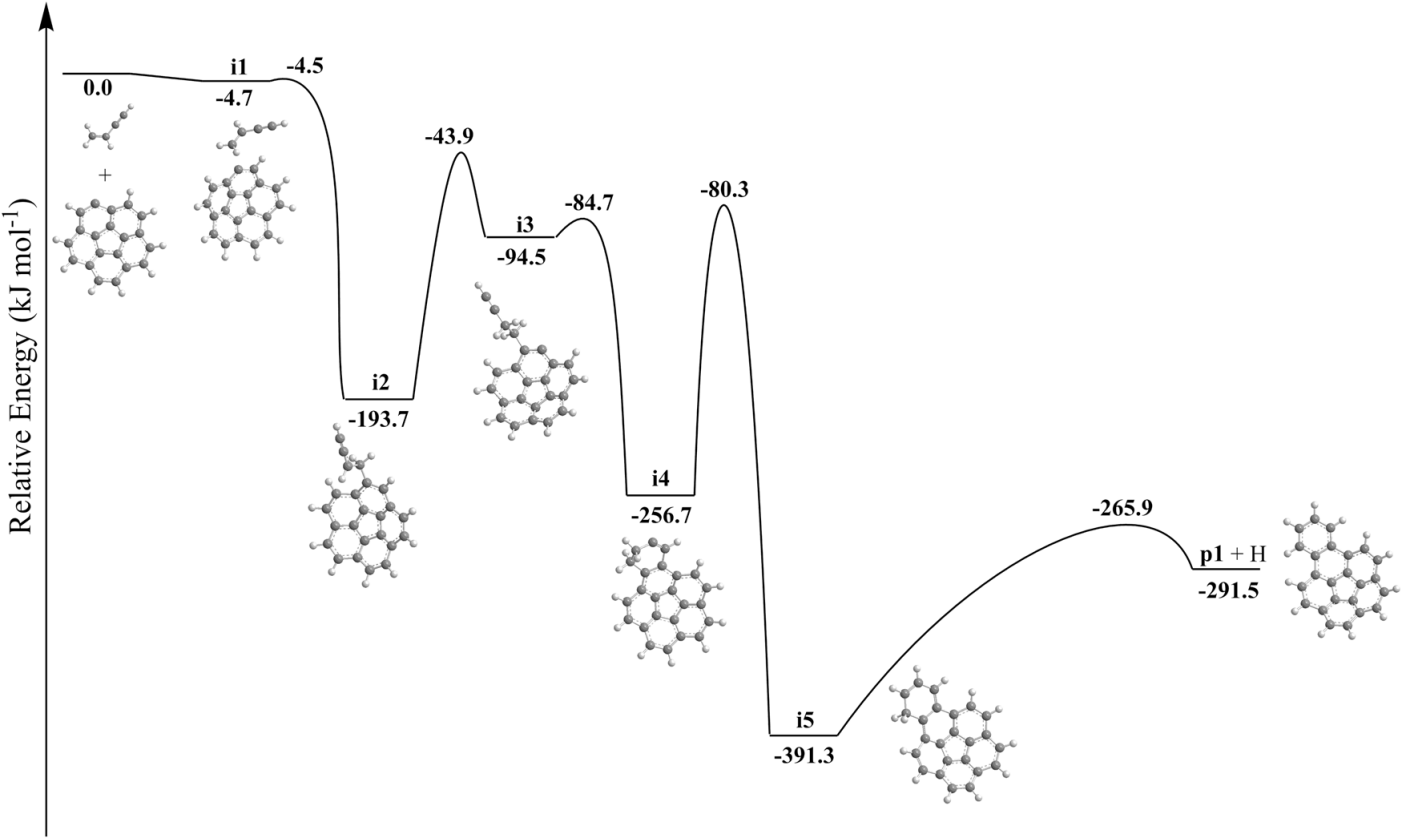
Potential energy diagram leading to benzocorannulene. Calculated potential energy diagram for the [C_20_H_9_]^•^ plus vinylacetylene reaction. Relative energies of various species calculated at the DLPNO-CCSD(T)/cc-pVDZ level of theory are given in kJ mol^−1^ with respect to the initial reactants. A full version of the diagram and the Cartesian coordinates are available in Supplementary Fig. 6 and Supplementary Data 1. Energies are calculated with a chemical accuracy of about 10 kJ mol^−1^. Carbon atoms are gray and hydrogen atoms are white.

The corannulenyl radical (C_20_H_9_^•^) first forms a van der Waals complex **i1** with vinylacetylene (C_4_H_4_) stabilized by 4.7 kJ mol^−1^ (Fig. 6). In order to form a covalent C–C bond between the vinylic terminal CH_2_ moiety and the radical site in C_20_H_9_^•^, the system needs to overcome a small barrier of 0.2 kJ mol^−1^ with respect to **i1**; the corresponding transition state is submerged with respect to the initial reactants since it resides 4.5 kJ mol^−1^ below their energy level thus resulting in an effectively barrierless formation of **i2** via C–C bond formation. This intermediate can eventually undergo ring annulation and hydrogen atom loss resulting in benzocorannulene (**p1**). In detail, this process involves migration of the hydrogen atom from the *ortho* position in the attacked aromatic ring in **i2** to the *β* carbon atom in the side chain (**i2** → **i3**), a facile six-membered ring closure (**i3**→ **i4**) via a barrier of only 10 kJ mol^−1^, a hydrogen atom shift in the newly formed six-membered ring from a CH_2_ group to the neighboring bare carbon atom (**i4** → **i5**), and elimination of a hydrogen atom from the remaining CH_2_ group accompanied by aromatization to **p1**. Several pathways to side-chained C_24_H_12_ isomers exist, but at 10 K these reactions are not competitive with the exoergic formation of benzocorannulene (C_24_H_12_) (−292 kJ mol^−1^) (Supplementary Fig. 6). Overall, as supported by kinetics calculations as detailed in Supplementary Note 4, the barrierless reaction to benzocorannulene represents a facile pathway even at temperatures of 10 K in cold molecular clouds; at elevated temperatures, **p1** is formed predominantly via hydrogen-assisted isomerization of the side-chained isomers 4-(1-buten-3-yne)corannulene and 4-(3-buten-1-yne)corannulene (Supplementary Fig. 8).

**Fig. 7:**
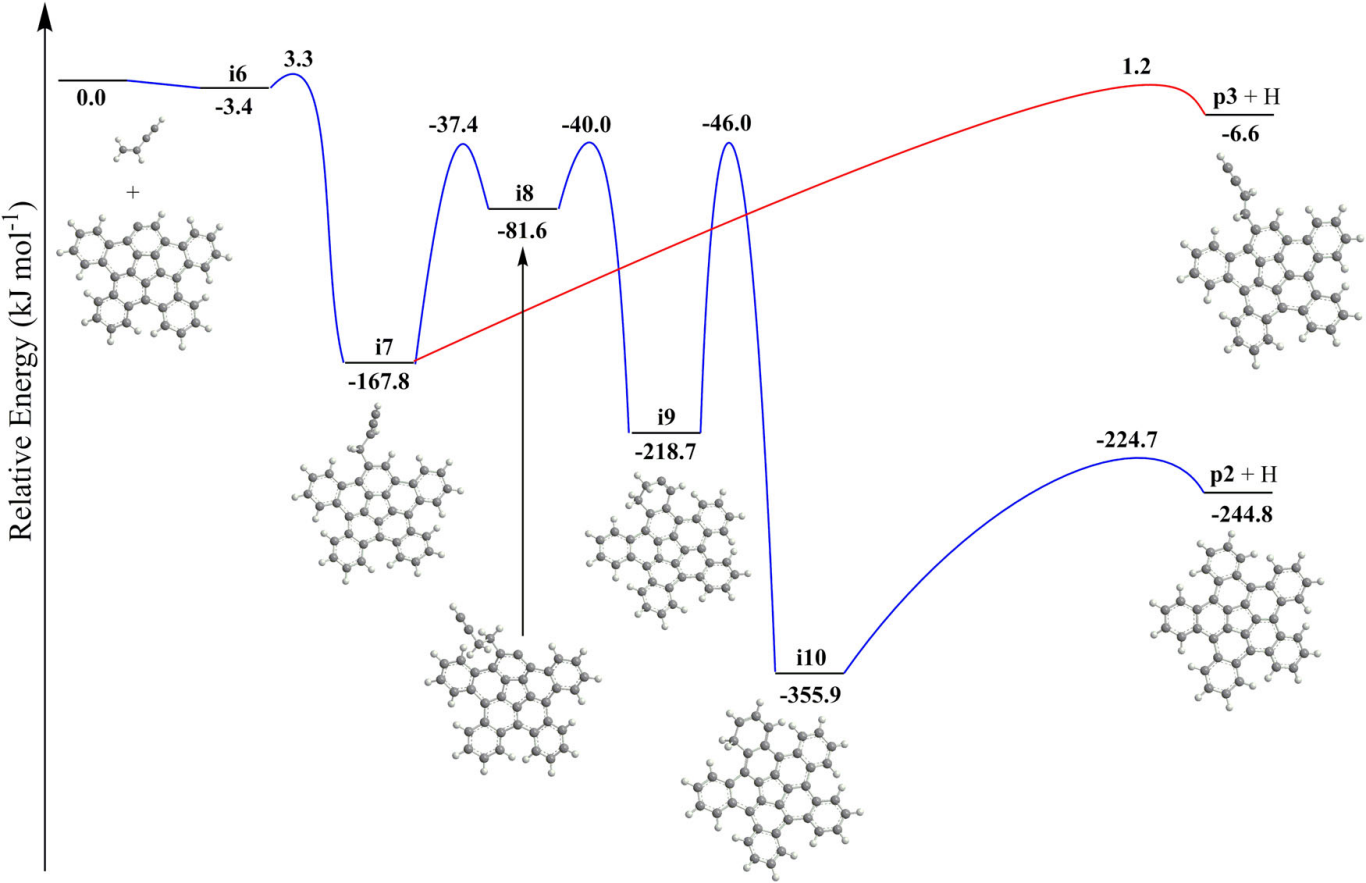
Potential energy diagram leading to pentabenzocorannulene. Calculated potential energy diagram for the most important channels of the [C_36_H_17_]^•^ plus vinylacetylene reaction. Relative energies of various species calculated at the DLPNO-CCSD(T)/cc-pVDZ level of theory are given in kJ mol^−1^ with respect to the initial reactants. A full version of the diagram and Cartesian coordinates are available in Supplementary Fig. 7 and Supplementary Data 2. Carbon atoms are gray and hydrogen atoms are white.

The experimental and theoretical results unambiguously demonstrate a facile formation of benzocorannulene (C_24_H_12_). Therefore, analogous benzannulation routes are feasible from benzocorannulene to pentabenzocorannulene (Fig. 2), where the ring annulation sequentially occurs on all five outside six-membered rings of the initial corannulene molecule. Note that in cold molecular clouds at 10 K, hydrogen abstraction reactions activating closed-shell species to radicals prior to their reactions with vinylacetylene are closed due to the inherent barriers of abstraction; however, the ultraviolet photon field present even deep inside cold molecular clouds and around planetary nebulae can photolyze corannulene leading via carbon-hydrogen bond rupture to the corannulenyl radical plus atomic hydrogen. Since the hydrogen abstraction and specifically vinylacetylene addition reactions proceed through benzannulation^46^ (Supplementary Table 1), here we focus on the potential energy diagram for the tetrabenzocorannulenyl radical (C_36_H_17_^•^) plus vinylacetylene (C_4_H_4_) reaction, i.e., the final ring annulation pathway (Fig. 7). The reactants can form a van der Waals complex **i6** stabilized by 3.4 kJ mol^−1^. Next, the formation of the covalently bound intermediate **i7** proceeds via a 6.7 kJ mol^−1^ barrier; this barrier is not submerged as the corresponding transition state resides 3.3 kJ mol^−1^ above the reactants’ zero energy level. The latter undergoes a hydrogen shift to **i8** followed by the six-membered ring closure to **i9**, a hydrogen migration to **i10**, and finally, hydrogen atom elimination to pentabenzocorannulene (C_40_H_12_, **p2**). The critical transition state on the pathway from **i7** to **p2** is the first hydrogen shift step with the transition state located 37 kJ mol^−1^ lower in energy than the separated reactants. This position is 7 kJ mol^−1^ higher than that for the corresponding **i2**→**i3** transition state in the reaction of the [C_20_H_9_]^•^ radical with vinylacetylene (Fig. 6). Also, the overall reaction exoergicity to produce **p2** decreases to 245 kJ mol^−1^ from 292 kJ mol^−1^ for the formation of **p1** and generally, the relative energies of the intermediates and transition states on the ring annulation pathway in the [C_36_H_17_]^•^ plus vinylacetylene reaction increase by up to 35–47 kJ mol^−1^ compared to [C_20_H_9_]^•^ plus vinylacetylene. This is also the case for the formation of **p3**, where the relative energies of the hydrogen loss transition state from **i7** and the product increase to 1 kJ mol^−1^ above and 7 kJ mol^−1^ below the reactants’ level.

**Fig. 8:**
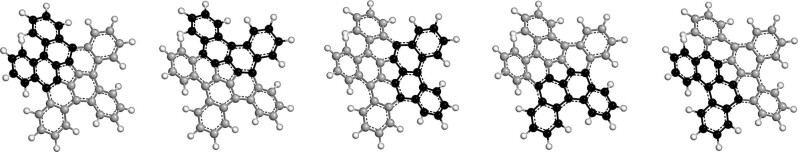
[4]-Helicene units in pentabenzocorannulene. Five overlapping [4]-helicene moieties in the structure of pentabenzocorannulene. Carbon atoms in each [4]-helicene unit are highlighted in black, the remaining carbon atoms are gray, and hydrogen atoms are white.

Pentabenzocorannulene is chiral, as are the intermediate di-, tri-, and tetrabenzocorannulenes all containing at least one [4]-helicene moiety in their structures. As illustrated in Fig. 8, pentabenzocorannulene contains five overlapping [4]-helicene units in its structure. Interestingly, the in-between vinylacetylene addition reactions do not have to have positive entrance barriers; we anticipate that the barriers remain submerged if hydrogen atom abstraction preceding vinylacetylene addition takes place at a zigzag edge but would be positive if hydrogen abstraction happens on an armchair edge of the intermediate benzocorannulenes. In addition, there is a multitude of different pathways from benzocorannulene to tetrabenzocorannulene depending on which hydrogen atom is abstracted at each stage (Fig. 2). According to the computed rate constants for the [C_36_H_17_]^•^ plus vinylacetylene reaction (Supplementary Fig. 8c), its kinetics are analogous to those of [C_20_H_9_]^•^ plus vinylacetylene. At the conditions relevant to combustion flames or circumstellar envelopes, the primary reaction would mostly form the side chain C_40_H_20_ isomers like **p3**, but their secondary, hydrogen-assisted isomerization would efficiently convert them into the much more thermodynamically favorable **p2**.

**Fig. 9:**
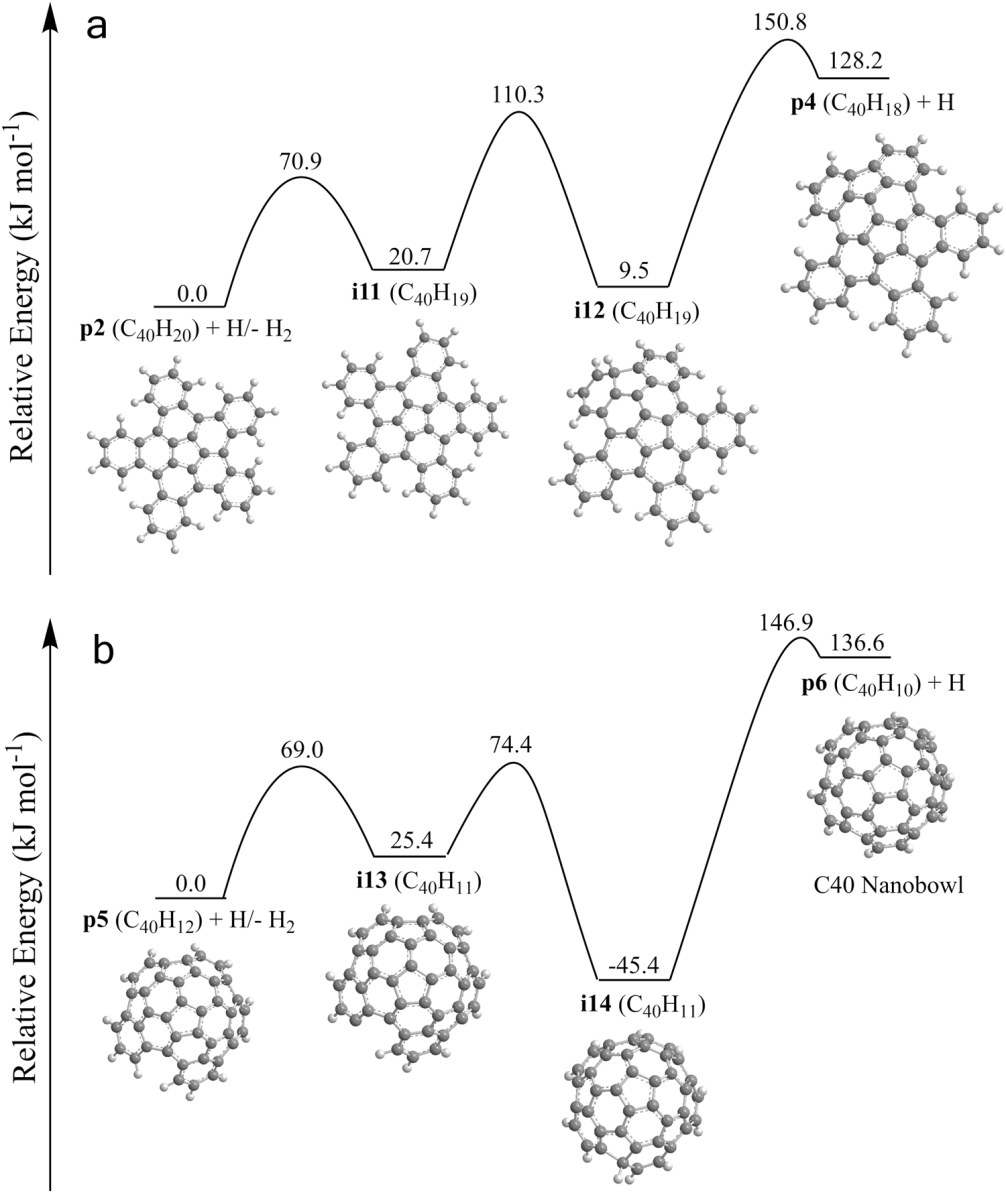
Potential energy diagram leading to the C40 nanobowl. Calculated potential energy diagrams for the conversion of pentabenzocorannulene (C_40_H_20_) to C_40_H_18_ (**a**) and C_40_H_12_ to the C40 nanobowl (C_40_H_10_) (**b**) via a hydrogen abstraction–cyclodehydrogenation mechanism. Relative energies of various species calculated at the DLPNO-CCSD(T)/cc-pVDZ level of theory are given in kJ mol^−1^ with respect to the initial reactants. A full version of the diagram for all five hydrogen atom abstraction–cyclodehydrogenation steps along with the Cartesian coordinates is available in Supplementary Fig. 10 and Supplementary Data 3 and 4. Carbon atoms are gray and hydrogen atoms are white.

Finally, we consider the fate of **p2** at high-temperature conditions in the presence of radicals, which can abstract hydrogen atoms, or of a strong vacuum ultraviolet photon field in deep space, which cleave the carbon-hydrogen bond. **p2** possesses five bay areas, which may undergo a five-membered ring closure following a hydrogen loss reaction either through hydrogen abstraction or photolysis. Thus, five consecutive hydrogen abstraction—five-membered ring closure—hydrogen atom loss (cyclodehydrogenation) processes may potentially lead to the C40 nanobowl (C_40_H_10_) (Fig. 9 and Supplementary Fig. 10) as shown in Eqs. 1–6.

1 $${{{{{{\rm{C}}}}}}}_{40}{{{{{{\rm{H}}}}}}}_{20}+{{{{{\rm{H}}}}}}\to {{{{{{\rm{C}}}}}}}_{40}{{{{{{{\rm{H}}}}}}}_{19}}^{{{\bullet }}}+{{{{{{\rm{H}}}}}}}_{2}\to {{{{{\rm{ring}}}}}}\; {{{{{\rm{closure}}}}}}\; {{{{{\rm{in}}}}}}\;{{{{{{\rm{C}}}}}}}_{40}{{{{{{\rm{H}}}}}}}_{19}\to {{{{{{\rm{C}}}}}}}_{40}{{{{{{\rm{H}}}}}}}_{18}+{{{{{\rm{H}}}}}}$$

2 $${{{{{{\rm{C}}}}}}}_{40}{{{{{{\rm{H}}}}}}}_{18}+{{{{{\rm{H}}}}}}\to {{{{{{\rm{C}}}}}}}_{40}{{{{{{{\rm{H}}}}}}}_{17}}^{{{\bullet }}}+{{{{{{\rm{H}}}}}}}_{2}\to {{{{{\rm{ring}}}}}}\; {{{{{\rm{closure}}}}}}\; {{{{{\rm{in}}}}}}\;{{{{{{\rm{C}}}}}}}_{40}{{{{{{\rm{H}}}}}}}_{17}\to {{{{{{\rm{C}}}}}}}_{40}{{{{{{\rm{H}}}}}}}_{16}+{{{{{\rm{H}}}}}}$$

3 $${{{{{{\rm{C}}}}}}}_{40}{{{{{{\rm{H}}}}}}}_{16}+{{{{{\rm{H}}}}}}\to {{{{{{\rm{C}}}}}}}_{40}{{{{{{{\rm{H}}}}}}}_{15}}^{{{\bullet }}}+{{{{{{\rm{H}}}}}}}_{2}\to {{{{{\rm{ring}}}}}}\; {{{{{\rm{closure}}}}}}\; {{{{{\rm{in}}}}}}\;{{{{{{\rm{C}}}}}}}_{40}{{{{{{\rm{H}}}}}}}_{15}\to {{{{{{\rm{C}}}}}}}_{40}{{{{{{\rm{H}}}}}}}_{14}+{{{{{\rm{H}}}}}}$$

4 $${{{{{{\rm{C}}}}}}}_{40}{{{{{{\rm{H}}}}}}}_{14}+{{{{{\rm{H}}}}}}\to {{{{{{\rm{C}}}}}}}_{40}{{{{{{{\rm{H}}}}}}}_{13}}^{{{\bullet }}}+{{{{{{\rm{H}}}}}}}_{2}\to {{{{{\rm{ring}}}}}}\; {{{{{\rm{closure}}}}}}\; {{{{{\rm{in}}}}}}\;{{{{{{\rm{C}}}}}}}_{40}{{{{{{\rm{H}}}}}}}_{13}\,\to {{{{{{\rm{C}}}}}}}_{40}{{{{{{\rm{H}}}}}}}_{12}+{{{{{\rm{H}}}}}}$$

5 $${{{{{{\rm{C}}}}}}}_{40}{{{{{{\rm{H}}}}}}}_{12}+{{{{{\rm{H}}}}}}\to {{{{{{\rm{C}}}}}}}_{40}{{{{{{{\rm{H}}}}}}}_{11}}^{{{\bullet }}}+{{{{{{\rm{H}}}}}}}_{2}\to {{{{{\rm{ring}}}}}}\; {{{{{\rm{closure}}}}}}\; {{{{{\rm{in}}}}}}\;{{{{{{\rm{C}}}}}}}_{40}{{{{{{\rm{H}}}}}}}_{11}\to {{{{{{\rm{C}}}}}}}_{40}{{{{{{\rm{H}}}}}}}_{10}+{{{{{\rm{H}}}}}}$$

6 $${{{{{{\rm{C}}}}}}}_{40}{{{{{{\rm{H}}}}}}}_{20}\to {{{{{{\rm{C}}}}}}}_{40}{{{{{{\rm{H}}}}}}}_{10}+5{{{{{{\rm{H}}}}}}}_{2}$$

The hydrogen abstraction barrier for C_40_H_20_ (71 kJ mol^−1^) is close to a typical value for the hydrogen abstraction reaction from an armchair PAH edge (69 kJ mol^−1^) and compares to 68 kJ mol^−1^ for a zigzag edge and 67 kJ mol^−1^ for benzene and naphthalene^47^; the hydrogen atom abstraction barriers remain in the narrow range of 69–75 kJ mol^−1^ for the complete reaction sequence (Supplementary Fig. 10). The hydrogen abstraction reaction to [C_40_H_19_]^•^ (**i11**) is 21 kJ mol^−1^ endoergic, which is lower than the typical endoergicity of 30 kJ mol^−1^ for such a reaction from an armchair edge of a PAH. At the next step, **i11** undergoes a five-membered ring closure at the affected bay area producing **i12**. This reaction stage is completed by a loss of the extra hydrogen from the newly created five-membered ring overcoming a 141 kJ mol^−1^ barrier and forming C_40_H_18_ (**p4**). The two-step unimolecular decomposition of **i11** via atomic hydrogen loss to **p4** is overall 108 kJ mol^−1^ endoergic and requires the highest barrier of 130 kJ mol^−1^ with respect to the initial reactants. Such energy demands can be easily accomplished at high temperatures with the entropy factor driving the reaction toward the cyclization followed by hydrogen atom loss, as supported by kinetic calculations (Supplementary Note 5). The corresponding values for the whole series of five cyclodehydrogenation reactions reveal narrow ranges in the intervals of 83–111 and 103–130 kJ mol^−1^, respectively (Fig. 9 and Supplementary Fig. 10).

Overall, the results of kinetics and equilibrium calculations (Supplementary Figs. 8 and 9) reveal that the forward cyclodehydrogenation reactions, while being endoergic, strongly dominate at high temperatures; this finding allows us to conclude that once **p2** is synthesized in the gas-phase, it can be efficiently converted into the C40 nanobowl (C_40_H_10_, **p6**) through a series of five consecutive hydrogen abstraction–five-membered-cyclodehydrogenation stages under high-temperature conditions as in combustion flames. Alternatively, ultraviolet photons can also cleave the carbon-hydrogen bond in deep space. Interestingly, the hydrogen atoms or alternative abstracting radicals would play a dual role in this mechanism; on one hand, they promote the hydrogen abstraction reactions, which activate the otherwise stable PAH molecules but, on the other hand, the increase in their concentration shifts the equilibrium in unimolecular decomposition of the radical PAH species in the reverse direction. Overall, the C_40_H_20_ → C_40_H_10_ + 5H_2_ reaction sequence catalyzed by hydrogen atoms can be classified as a subtype of hydrogen atom removal–cyclodehydrogenation reaction sequences.

Once a hydrogen atom is lost from the armchair edge of the C_40_H_20_–C_40_H_12_ molecules, the most favorable reaction pathway is the five-membered ring closure followed by a hydrogen loss as demonstrated above. The only alternative pathway is a hydrogen atom loss from the *ortho* position with respect to the radical to form an aromatic C–C triple bond forming an *o*-benzyne-like structure which is endoergic by about 335 kJ mol^−1^; this energy is much higher than the energy needed for the cyclodehydrogenation reaction to occur. Clearly, the reaction scenario depends on which hydrogen atom is abstracted at the initial step of the reaction sequence. The hydrogen atom abstraction from an armchair edge unequivocally leads to cyclodehydrogenation (Supplementary Fig. 10). Alternatively, hydrogen atom abstraction from outer carbon atoms of six-membered rings cannot be followed by cyclodehydrogenation directly. Here, possible scenarios would include bimolecular reactions of the radical with other species present, for example, yet another benzannulation via vinylacetylene addition, or acetylene addition—hydrogen atom elimination, or propargyl addition. This would result in a different growth pathway not leading to **p6**. The reaction can also proceed by hydrogen atom migration from the neighboring carbon atom at an armchair edge followed by cyclodehydrogenation. Such migrations have been demonstrated to be fast and highly probable at high temperatures by our recent kinetic Monte Carlo simulations of PAH growth^48^. Also, in the presence of other radicals in the system, e.g., hydroxyl (OH), they can abstract hydrogen atoms from the C_40_H_x_ molecules with an even higher rate constant^47^. Considering that the equilibrium constant for cyclodehydrogenation reactions grows with a decrease of the concentration of hydrogen atoms, the use of the radicals such as hydroxyl for the activating hydrogen atom abstraction step would further facilitate the reaction in the forward direction toward the formation of the nanobowl molecule.

A combination of a molecular beam experiment and isomer-selective detection of the reaction products with electronic structure and kinetics calculations allowed us to unravel the formation mechanism of benzocorannulene (C_24_H_12_) via ring annulation. Exploiting this prototype of benzannulation, we computationally elucidated viable pathways to the gas-phase synthesis of the C40 nanobowl (C_40_H_10_) via five consecutive benzannulation steps to pentabenzocorannulene (C_40_H_20_) followed by five five-membered ring closures at the armchair edges at high temperatures yielding the C40 nanobowl. The benzannulation steps can alternate with the hydrogen abstraction–cyclodehydrogenation steps; the latter becomes possible as soon as two neighboring outside six-membered rings of corannulene have been benzannulated. As a molecular building block of fullerene (C_60_), we anticipate that such mechanisms could be common in the formation and growth of buckyballs and carbon nanotubes not only in combustion flames, but also in circumstellar envelopes and planetary nebulae of carbon-rich stars, which feature similar temperatures. At any particular environment, the mechanism is governed by the presence and abundance of hydrocarbon species required for the addition steps (vinylacetylene, C_4_H_4_) and radicals (H, OH) driving the hydrogen atom abstraction steps activating closed-shell PAH molecules partaking in the growth process.

## Methods

### Experimental

The experiments were conducted at the X04DB beamline of the Swiss Light Source (SLS) at the Paul Scherrer Institute (PSI) utilizing a resistively heated silicon–carbide (SiC) chemical microreactor coupled to a molecular beam apparatus operated with a double velocity map imaging (VMI) photoelectron photoion coincidence spectrometer^41,49–54^. This setup allows the exploration of molecular mass growth processes to aromatic molecules in situ via elementary reactions of pyrolytically generated aryl radicals. In detail, bromocorannulene (C_20_H_9_Br; synthesized in house) was sublimed at 443 ± 1 K in a high-vacuum oven in the source chamber, and was seeded in vinylacetylene (C_4_H_4_; 5% in helium, Applied Gas Inc.) at a backing pressure of 100 mbar. The gas mixture was introduced through a 200 µm diameter nozzle into a 35 mm long SiC tube with 1 mm inner diameter at a heated length of 20 mm. Corannulenyl radicals (C_20_H_9_^•^) were created in situ via pyrolysis of bromocorannulene at a reactor temperature of 1200 ± 100 K measured by a type C thermocouple. These radicals react with the vinylacetylene reactant/seeding gas to form the products, which exit the microreactor and attain supersonic expansion before passing through a 2 mm diameter skimmer to the experimental chamber, which houses the photoelectron photoion coincidence (PEPICO) spectrometer. Here, the products were photoionized by quasi-continuous synchrotron vacuum ultraviolet (VUV) radiation tuned from 6.50 to 9.00 eV in 0.02 eV steps. VUV single-photon ionization represents a fragment-free technique and therefore is considered a soft ionization method^55,56^. The ions and electrons formed via photoionization were extracted in opposing directions by a 218 V cm^−1^ electric field and were each imaged on separate position-sensitive delay-line anode detectors (Roentdek DLD40). The photoelectrons served as a time zero for the time-of-flight (TOF) measurement of the coincident photoion. PIE curves, which report ion counts at a well-defined mass-to-charge ratio (*m*/*z*) as a function of photon energy, were obtained by integrating the signal at the specific *m*/*z* associated with the species of interest. Photoion mass-selective threshold photoelectron (ms-TPE) spectra were obtained from the same scan by selecting only electrons with <10 meV kinetic energy, in coincidence with photoions in the mass range of interest. The hot electron signal, with an off-axis momentum, was subtracted from the threshold (low kinetic energy less than 10 meV) electron signal by the procedure of Sztaray et al.^57^. ms-TPES obtained from hot pyrolysis reactors often suffer from spectral broadening due to insufficient expansion cooling. However, by integrating solely the room temperature background velocity component in the ions’ VMI, a reduction of hot band transitions can be achieved and gives rise to room temperature ms-TPES^58^. All spectra were normalized to the photon flux and corrected by 11 meV due to the Stark shift at a constant electric field of 218 V cm^−1^^59^. PIE and ms-TPE calibration curves of benzocorannulene (C_24_H_12_; synthesized in house) were collected for comparison to the reactive experiments by subliming benzocorannulene at 473 ± 1 K in the high-vacuum oven and seeding it in helium (He; 99.996%, PanGas) at a backing pressure of 130 mbar without pyrolysis.

### Computational

The molecular parameters including geometries, rotational constants, and vibrational frequencies of the reactants, products, along with intermediates and transition states for the reactions of corannulenyl and tetrabenzocorannulenyl radicals with vinylacetylene proceeding on the [C_24_H_13_]^•^ and [C_40_H_21_]^•^ potential energy surfaces (PES), as well as for the hydrogen atom abstraction–cyclodehydrogenation reactions involving C_40_H_20_ and C_40_H_12_ were calculated using the density functional theory (DFT) B3LYP/6-311G(d,p) level of theory^60–62^. Using the optimized geometries, single point energies were refined within more accurate wavefunction-based theoretical methods. For the smallest [C_20_H_9_]^•^ plus C_4_H_4_ system, we employed coupled cluster CCSD(T) and second-order Møller–Plesset perturbation theory MP2 calculations, with the final G3(MP2,CC) energy being computed as^63^ E[G3(MP2,CC)] = E[CCSD(T)/6-31G(d)] + E[MP2/G3(Large)] – E[MP2/6-31G(d)] + ZPE[B3LYP/6-311G(d,p)]. The model chemistry G3(MP2,CC) approach provides chemical accuracy of 0.01–0.02 Å for bond lengths, 1–2° for bond angles, and 3–6 kJ mol^−1^ for relative energies of hydrocarbons, their radicals, reaction energies, and barrier heights in terms of average absolute deviations. In addition, the energies were recalculated using domain-based local pair-natural orbital singles and doubles coupled cluster method perturbatively included connected triple excitations (DLPNO-CCSD(T))^64^ with Dunning’s cc-pVDZ and cc-pVQZ basis sets^65^. This allowed us to compare the performance of DLPNO-CCSD(T) for PAH growth reactions with the well-established model chemistry method and also to evaluate the convergence of DLPNO-CCSD(T) results with respect to the basis set. Two methods, ONIOM2 {G3(MP2,CC):B3LYP/6-311G(d,p)}^65–67^ and DLPNO-CCSD(T)/cc-pVDZ, were used for the energy refinement of various structures on the C_40_H_21_ PES accessed by the tetracorannulenyl plus vinylacetylene reaction. The ONIOM2 approach has recently been shown to provide accurate energies for large PAH systems with a careful choice of model systems^66,67^. Here, as compared to the real [C_40_H_21_]^•^ radical, the model system excluded four extra benzo rings around the corannulene core in the tetracorannulenyl radical with the dangling valences of the outside carbon atoms being saturated by hydrogens. Thus, the stoichiometry of the model system was only [C_24_H_13_]^•^ making the G3(MP2,CC) calculations within the ONIOM2 scheme affordable. Finally, for hydrogen atom abstraction and cyclodehydrogenation reactions involving C_40_H_20_ and C_40_H_12_ we used the DLPNO-CCSD(T)/cc-pVDZ method judging from its performance for [C_24_H_13_]^•^ and [C_40_H_21_]^•^. The choice of an appropriate model system for ONIOM calculations here is challenging because the entire buckled carbon skeleton is involved in the cyclization reactions.

Adiabatic ionization energies of possible C_24_H_12_ products of the corannulenyl plus vinylacetylene reactions were computed using the G3(MP2,CC)//B3LYP/6-311G(d,p) method with zero point energy (ZPE) corrections; the expected accuracy, in this case, is ±0.05 eV. Moreover, ionization Franck-Condon factors at 0 K needed for a comparison with the experimental ms-TPE spectra were calculated using B3LYP/cc-pVTZ-optimized geometries and corresponding vibrational frequencies of the neutral and cationic species using the methodology implemented by Barone and co-workers^68^. For benzocorannulene, Franck-Condon factors were additionally reevaluated at elevated temperatures utilizing the ezSpectra code by Krylov et al.^69^. Electronic excitation energies of the benzocorannulene cation were estimated within the time-dependent (TD)-DFT method^70^ with the ωB97XD functional^71^ and cc-pVTZ basis set^64^. All the ab initio and DFT calculations were carried out using the GAUSSIAN 16^72^ (B3LYP, ωB97XD, and evaluation of Franck-Condon factors at zero Kelvin), MOLPRO 2021^73^ (CCSD(T) and MP2), and ORCA^74^ (DLPNO-CCSD(T)) quantum chemistry program packages.

Finally, pressure- and temperature-dependent rate constants and product branching ratios for the corannulenyl/tetrabenzocorannulenyl plus vinylacetylene reactions and for cyclodehydrogenation of [C_40_H_19_]^•^ and [C_40_H_11_]^•^ produced by hydrogen atom abstractions from pentabenzocorannulene C_40_H_20_ and C_40_H_11_, respectively, were assessed using the Rice–Ramsperger–Kassel–Marcus Master Equation (RRKM-ME) theoretical approach utilizing the MESS software package^75^. Here, partition functions for local minima and transition states were computed within the Rigid-Rotor, Harmonic-Oscillator (RRHO) model. Tunneling corrections were included using asymmetric Eckart potentials. The Lennard-Jones parameters ε and σ were estimated based upon the molecular mass of the intermediates involved in each particular reaction as proposed by Wang and Frenklach^76^ and the parameters for the nitrogen bath gas were taken from Vishnyakov et al.^77^. The exponential down model was employed to treat the collisional energy transfer in ME, with the temperature dependence of the range parameter *α* for the deactivating wing of the energy transfer function expressed as

7 $$\alpha\left(T\right)=\alpha_{300}{\left(\frac{T}{300K}\right)}^{n}.$$

where the values of *n* = 0.85 and *α*_300_ = 247 cm^−1^ proposed as universal for hydrocarbons were adopted^78^. Supplementary Data 1–4 include input files for the RRKM-ME calculations using MESS, which incorporate optimized Cartesian coordinates and computed vibrational frequencies for all structures considered in this work.

## Supplementary information

Supplementary Information

Description of Additional Supplementary files

Supplementary Data 1

Supplementary Data 2

Supplementary Data 3

Supplementary Data 4

## Data Availability

All data generated in this study are available in the main text and the supplementary materials.
