# Supplementary material for: Gas phase synthesis of the C40 nano bowl C40H10
Source: Nat Commun. 2023 Mar 18;14:1527. doi: 10.1038/s41467-023-37058-y (PMC10024697; doi:10.1038/s41467-023-37058-y)
Supplement: Supplementary file 1 — Supplementary Information [file 41467_2023_37058_MOESM1_ESM.pdf]

# Supplementary Information

## Gas Phase Synthesis of the C40 Nano Bowl $C_{40}H_{10}$

Tuli et al.

**Supplementary Note 1: Theoretical methods performance comparison in potential energy diagram calculations.**

For the corannulenyl reaction considered here, there is a close agreement between the relative energies computed by our standard G3(MP2,CC) level and those obtained at DLPNO-CCSD(T) both with the cc-pVDZ and cc-pVQZ basis sets (see Supplementary Fig. 6 and Supplementary Table 1). The differences are normally within 3 kJ mol$^{-1}$ for the ring annulation pathway but somewhat larger, up to 11 kJ mol$^{-1}$, for the 4-(1-buten-3-yne)corannulene and 4-(3-buten-1-yne)corannulene products and the respective hydrogen atom loss transition states. Also, the DLPNO-CCSD(T) results with the cc-pVDZ and cc-pVQZ basis sets are very close to one another with few exceptions (**i4** and **p1**), where the deviation is 8-9 kJ mol$^{-1}$. This comparison indicates that the chemical accuracy of about 10 kJ mol$^{-1}$ can be normally achieved for molecules of this type employing the DLPNO-CCSD(T)/cc-pVDZ level of theory opening the opportunity to carry out accurate calculations for much larger systems than those treatable by the model chemistry G3/G4-type schemes. One can also see that the accuracy of DFT relative energies is not sufficient, with discrepancies from the more accurate results often exceeding 40 kJ mol$^{-1}$ and not being consistent.

The calculations of the $[C_{36}H_{17}]^{\bullet}$ + $C_4H_4$ potential energy diagram were additionally carried out at the ONIOM2(G3(MP2,CC):B3LYP/6-311G(d,p)) level of theory, where the central corannulene moiety was chosen as the model system, with four outside six-membered rings removed and replaced by H atoms saturating the dangling bonds. The agreement between the DLPNO-CCSD(T)/cc-pVDZ and ONIOM relative energies is reasonably close, with the average absolute deviation of 5.8 kJ mol$^{-1}$ (Supplementary Fig. 7 and Supplementary Table 1). The largest discrepancy of 12.6 kJ mol$^{-1}$ is found for **i9** where the ONIOM value underestimates the DLPNO-CCSD(T) calculated well depth. Nevertheless, it is evident that both DLPNO-CCSD(T)/cc-pVDZ and ONIOM methods represent viable alternative for calculations of large PAH systems with nearly chemical accuracy. Again, the deviations of B3LYP energies from the more accurate results are significantly higher.

**Supplementary Note 2: Alternative pathways in the $[C_{20}H_9]^•$ + $C_4H_4$ reaction**.

In a competing reaction pathway, **i2** can directly dissociate to *trans*-4-(3-buten-1-yne)corannulene **p9** by splitting a hydrogen atom from the $\alpha$ carbon in the side chain (Supplementary Fig. 6). The transition state for the hydrogen atom loss and the **p9** + H products reside at 22 and 41 kJ mol$^{-1}$ lower in energy than the reactants (in this Section we discuss the values obtained at the G3(MP2,CC) level of theory which was routinely used for all structures). Also, **i2** can rearrange to **i16** by shifting the corannulene moiety over the $CH_2$-CH bond in the side chain overcoming a 104 kJ mol$^{-1}$ barrier. Intermediate **i16** can be formed from the initial complex **i1** by linking the radical site in $[C_{20}H_9]^•$ with the C2 atom of vinylacetylene via a transition state positioned 5 kJ mol$^{-1}$ above the reactants. Next, **i16** can lose the hydrogen atom from the attacked C2 carbon forming the **p8** product 3-(3-buten-1-yne)corannulene exoergic by 36 kJ mol$^{-1}$ via a transition state lying 3 kJ mol$^{-1}$ above the reactants. In an alternative pathway, **i16** can feature a shift of the terminal side chain CH group over the CH-$CH_2$ bond producing a three-membered ring in **i17**, with the following ring opening rendering the **i18** intermediate with a $CHCH_2CCH$ side chain attached to the corannulenyl radical. Note that **i18** can be also achieved directly from **i2** by a 1,2-H shift in the side chain but via a significantly higher barrier of 188 kJ mol$^{-1}$ relative to **i2**. The **i16** $\rightarrow$ **i17** $\rightarrow$ **i18** isomerization is expected to be fast as it involves rather low ring closure/opening barriers. Subsequently, **i18** can dissociate to both *trans*- and *cis*-conformers of 4-(3-buten-1-yne)corannulene via transition states located 17 and 18 kJ mol$^{-1}$ below the separated reactants ($[C_{20}H_9]^•/C_4H_4$), respectively. The *cis*-4-(3-buten-1-yne)corannulene conformer is 7 kJ mol$^{-1}$ higher in energy than the *trans*-conformer. Vinylacetylene can also add to the corannulenyl radical by its acetylenic C4 atom producing the intermediate **i15**. The pathway to the latter from the van der Waals complex **i1** proceeds through a low barrier of ~7 kJ mol$^{-1}$, which is submerged by 0.4 kJ mol$^{-1}$ relative to the reactants. In turn, **i15** can dissociate by losing the hydrogen atom from C4 forming 4-(1-buten-3-yne)corannulene **p7** residing 49 kJ mol$^{-1}$ below the reactants via a transition state at 28 kJ mol$^{-1}$ lower in energy than the reactants.

Besides the vinylacetylene addition pathways described above and in the main text, the $[C_{20}H_9]^•$ + $C_4H_4$ reaction can proceed by direct hydrogen atom abstraction from vinylacetylene by the corannulenyl radical forming *i*- and *n*-$C_4H_3$ plus $C_{20}H_{10}$ (corannulene) via respective barriers of 16 and 32 kJ mol$^{-1}$. The abstraction reactions are calculated to be 51 kJ mol$^{-1}$ exoergic for *i*-

$C_4H_3$ and 1 kJ mol$^{-1}$ endoergic for *n*-$C_4H_3$. Note that other reaction pathways (e.g., vinylacetylene addition by C3 carbon atom or a lengthy isomerization pathway leading from **i15** to the **p1** + H products) are also possible as described in great detail for the prototype phenyl plus vinylacetylene reaction[1], but in this present study, we focus only on the channels which were proved to be most significant in this reaction class and lead to the experimentally observed products of the $[C_{20}H_9]^{\bullet}$ plus $C_4H_4$ reaction.

**Supplementary Note 3: Comparison of vinylacetylene addition reactions to various PAH radicals**.

It is informative to compare the corannulenyl plus vinylacetylene potential energy diagram computed here with those for the reactions of other PAH radicals with $C_4H_4$[2–4] focusing on the most relevant, for PAH growth, ring annulation channel and the competitive H loss pathways (Table S1). There is a large similarity of the relative energies computed here and in the previous works, especially with the 2-tetracenyl + $C_4H_4$ reaction channels leading to benzo[a]tetracene and its **p9**- and **p7**-like isomers[3]. The differences for most species do not exceed 5 kJ mol$^{-1}$ and the largest one, 23 kJ mol$^{-1}$, is observed for the six-membered ring closure transition state **i3-i4**. As compared to the smaller reacting radicals, phenyl and 1-naphthyl, corannulenyl has small but consistent stabilization of all intermediates, transitions states, and the benzannulated product along the ring annulation pathway. Also peculiar is that the barrier for vinylacetylene addition by the acetylenic C4 atom is clearly reduced for corannulenyl as compared to the other PAH radicals previously studied and even becomes slightly submerged at the G3(MP2,CC) level, while remaining marginally positive at DLPNO-CCSD(T). Generally, Table S1 demonstrates that the ring annulation reactions involving PAH radicals proceed by similar mechanisms although their quantitative details may somewhat vary depending on the size and shape of the reacting radical and the final expanded PAH product.

**Supplementary Note 4: Kinetics of the $[C_{20}H_9]^•$ plus $C_4H_4$ Reaction.**

The multichannel kinetics of benzannulation of aryl radicals with vinylacetylene is rather complex as multiple products can be formed, with their relative yields strongly depending on temperature and pressure. The formation of the most thermodynamically favorable benzannulated product, benzocorannulene, is strongly preferred at low temperatures and very low pressures which would exclude collisional stabilization of intermediate potential wells. As the pressure increases, the dominating low-temperature products, up to at least 1,000 K, are stabilized $[C_{24}H_{13}]^•$ intermediates including **i15**, **i2**, **i4**, and **i18**. At high temperatures relevant to the present experiment as well as to the conditions in combustion flames and circumstellar envelopes, the formation of the side-chained $C_{24}H_{12}$ isomers, such as **p7**, **p9**, and **p10**, is preferred (Supplementary Fig. 8(a)). The rate constant to produce **p1** at 1,200 K and 0.03 atm, which represents typical conditions inside the microreactor, is reasonably high, $3.4\times10^{-14}$ $cm^3$ $molecule^{-1}$, but the relative yield of **p1** in the primary reaction is predicted to be low as compared to **p7**, **p9**, and **p10**. However, the **p7**, **p9**, and **p10** isomers of $C_{24}H_{12}$, in addition to their mutual hydrogen-assisted isomerization and the reversal back to the initial reactants, can also efficiently convert to the much more stable **p1** species in the secondary reactions with H atoms (Supplementary Fig. 8(b)). For instance, the calculated rate constants for the **p10**/**p9** + H $\rightarrow$ **p1** + H reactions are on the order of $3\times10^{-13}$ $cm^3$ $molecule^{-1}$ under the reactor conditions. Although not particularly explored here due to the complexity of the channels involved, based on the analogy with phenyl plus vinylacetylene, the secondary **p7** + H reaction is anticipated to mostly produce the reactants as well as **p9** + H and **p1** + H. Our simulations of the chemical and physical processes inside the microreactor for phenyl plus vinylacetylene[1] have demonstrated that the analogs of **p9** and **p7** predominantly form first in the primary reaction and then are eventually converted to **p1** through secondary H-assisted reactions. A similar behavior might explain the formation of benzocorannulene as the prevailing product exiting the microreactor and identified in the present experiment at elevated temperatures. However, at conditions resembling cold molecular clouds (10 K), **p1** can be primarily formed via reaction of corannulenyl plus vinylacetylene.

**Supplementary Note 5: Kinetics of cyclodehydrogenation reactions.**

The kinetics of hydrogen abstraction from PAH molecules is well known[5] and this process is firmly established to play an important role in the growth of carbonaceous species and nanostructures[6]. Therefore, here we focus on unimolecular decomposition of the $[C_{40}H_{19}]^{•}$ and $[C_{40}H_{11}]^{•}$ radicals via cyclodehydrogenation to demonstrate that this process is indeed favorable at high temperatures. Supplementary Fig. 9 shows calculated reaction rate constants in the forward and reverse directions and equilibrium constants. The decomposition of $[C_{40}H_{19}]^{•}$ **i11** occurring through the five-membered ring closure followed by hydrogen loss is fast, with the rate constant at 1 atm reaching the values of $2.0\times10^{5}$ and $3.0\times10^{7}$ $s^{-1}$ at 1000 and 1500 K, respectively. **i11** survives as a distinct chemical species up to 1125, 1500, and 1800 K at the pressures of 0.03, 1, and 10 atm, respectively, and fully equilibrates (merges) with its decomposition product $C_{40}H_{18}$ **p4** at higher temperatures. A slightly different kinetic behavior is predicted for $[C_{40}H_{11}]^{•}$. Here, the **i13** intermediate is significantly less stable than **i14** being separated from the latter by a rather low barrier. The rate constant for the **i13** → **i14** isomerization is very fast even at 500 K ($1.7\times10^{7}$ $s^{-1}$) and hence **i13** merges with **i14** at or above the temperatures of 500, 600, and 800 K at 0.03, 1, and 10 atm. Therefore, we considered rate constants for unimolecular decomposition of **i14** (Supplementary Fig. 9(b)). Those are also high, amounting to $1.2\times10^{4}$ and $1.9\times10^{7}$ $s^{-1}$ at 1000 and 1500 K, respectively, at the atmospheric pressure. **i14** equilibrates with $C_{40}H_{10}$ **p6** + H at temperatures above 1250, 1500, 1750, and 2000 K at the pressures of 0.03, 1, 10, and 100 atm, respectively. Thus, $[C_{40}H_{11}]^{•}$ is more stable than $[C_{40}H_{19}]^{•}$, but its lifetime with the respect to the hydrogen atom loss is expected to be on a sub-microsecond scale at 1500 K and higher temperatures. On the other hand, the reverse bimolecular rate constants for $C_{40}H_{18}$ + H and $C_{40}H_{10}$ + H are also fast (Supplementary Fig. 9(c)), in the range of 2-3$\times10^{-12}$ $cm^{3}$ $molecule^{-1}$ $s^{-1}$ in the 1000-1500 K temperature interval at 1 atm for the former and 1-2$\times10^{-10}$ $cm^{3}$ $molecule^{-1}$ $s^{-1}$ for the latter. Those values are significantly higher than a competitive per site hydrogen abstraction[5] from an armchair PAH edge. All this is indicative of rapid equilibration between the radicals and hydrogen loss products if the hydrogen atoms remain available for the reverse reaction and are not consumed by other processes. It is therefore informative to consider the equilibrium constants, which depend on the mole fraction $x_{H}$ of available hydrogen atoms (Supplementary Fig. 9(d)). In order to test this hypothesis, we computed $K_{eq}$ at two hydrogen mole fraction values typical in

combustion flames, 0.0004 and 0.01[7]. We found that the **i11** ⇆ $C_{40}H_{18}$ + H and **i13** ⇆ $C_{40}H_{18}$ + H equilibria rapidly shift in the forward direction with increasing temperature exceeding unity at 800 and 1000 K for $[C_{40}H_{19}]^{\bullet}$ and at 900 and 1125 K for $[C_{40}H_{11}]^{\bullet}$ respectively at the $x_H$ values of 0.0004 and 0.01. At a typical combustion temperature of 1500 K, the values of $K_{eq}$ rise to $4.3\times10^3$ and $1.7\times10^2$ for $[C_{40}H_{19}]^{\bullet}$ and to $2.1\times10^3$ and $8.3\times10^1$ for $[C_{40}H_{11}]^{\bullet}$ and the forward reactions dominate even stronger at higher temperature.

## Supplementary Methods

**Synthesis of bromocorannulene.** Synthesis of bromocorannulene was carried out using the procedure given by Xu et al[8]. Briefly, to a 50 mL round-bottom flask, corannulene (1.0 g, 4.0 mmol) and *N*-bromosuccinimide (NBS) (712.0 mg, 4.0 mmol) were dissolved in 20 mL dichloroethane, then $BF_3 \cdot Et_2O$ (0.2 ml, 1.6 mmol) was added. The mixture was stirred at r.t. for 12 h and then quenched by 50 mL 10 % NaOH solution and extracted by DCM (2×50 mL). The organic layer was gathered and washed with water (2×50 mL) and dried using anhydrous $Na_2SO_4$. The solvent was evaporated under vacuum and a crude product (total weight: 1.21 g) was obtained. The conversion rate (79.1%) and yield of bromocorannulene (69.3 %, 87.5 % b.r.s.m.) was determined by analytical HPLC. The crude reaction mixture of corannulene, bromocorannulene, and dibromocorannulene was packed on a short silica plug and separated into two portions (portion a: total weight 515 mg, 90 % bromocorannulene/ 10 % dibromocorannulene; portion b: total weight 685 mg, 30 % corannulene/ 65 % bromocorannulene/ 5 % dibromocorannulene). Portions a and b were then dissolved in HPLC grade DCM and mixed with kieselguhr, respectively, before undergoing further separation using Combiflash-Rf. The separation process obtained 955 mg 98 % pure bromocorannulene in total, as well as recovered 200 mg 98 % pure corannulene. $^1$H-NMR data of bromocorannulene is consistent with reported values.

**Synthesis of benzocorannulene C.** Benzocorannulene has been synthesized from bromocorannulene **A**, which in turn has been prepared from corannulene by reported bromination protocol[9,10]. The Pd-catalyzed Stille cross-coupling reaction of **A** with $Bu_3Sn$(vinyl) in toluene at 100 ºC for 3 h afforded vinylcorannulene **B**[3] (52%; Supplementary Fig. 1). Subsequent reaction of **B** with nitroethanol in the presence of phthalic anhydride in *o*-dichlorobenzene at 180 ºC for 3 days provided **C**[3] (28%).

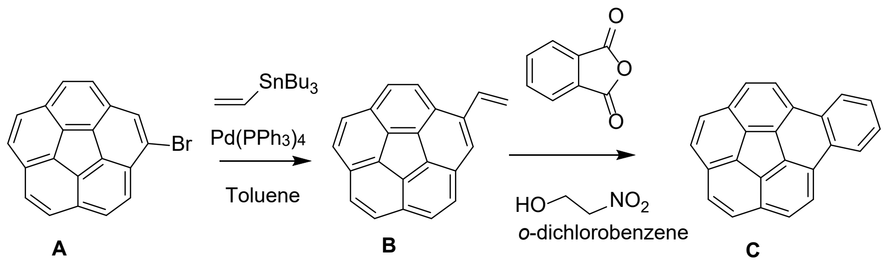


**Supplementary Fig. 1.** Synthesis of **C**

**1-Vinylcorannulene** (**B**). The **A**[1] (198 mg, 0.6 mmol) was dissolved in dry toluene (10 mL) in a flame-dried flask. Then $Pd(PPh_3)_4$ (23.2 mg, 0.02 mmol) and $Bu_3Sn$(vinyl) (210 µL, 227 mg, 0.72 mmol) were added at rt. The reaction mixture was heated at 110 °C and stirred for 5 h [progress of the reaction was monitored by TLC (hexane)]. Volatiles were evaporated and the residue was column chromatographed (5 → 10% EtOAc/hexane) to give **B**[9,10] (86 mg, 52%) as a light yellow powder: $^1$H NMR (400 MHz, $CDCl_3$) $\delta$ 5.60 (dd, $J$ = 10.8, 1.2 Hz, 1H), 6.13 (dd, $J$ = 17.2, 1.2 Hz, 1H), 7.36 (ddd, $J$ = 17.6, 10.8, 1.2 Hz, 1H), 7.74–7.88 (m, 8H), 8.08 (d, $J$ = 8.8 Hz, 1H); $^{13}$C NMR (101 MHz, $CDCl_3$) $\delta$ 118.24, 122.21, 124.46, 125.66, 127.01, 127.10, 127.17, 127.22, 127.37, 127.43, 127.57, 129.05, 130.79, 130.89, 130.94, 131.05, 134.81, 134.83, 135.54, 135.64, 135.79, 136.02, 136.35, 137.88.

**Benzocorannulene** (**C**). Compound **B** (80 mg, 0.29 mmol) was dissolved in mixture of o-dichlorobenzene (15 mL) and nitroethanol (1.0 mL, 1.27 g, 13.9 mmol) in a pressurized flask. Phthalic anhydride (2.1 g, 14.2 mmol) was added at rt. The reaction mixture was heated at 180 °C and stirred for 3 days. Volatiles were evaporated and the residue was extracted with EtOAc. The organic layer washed with 1. 0 M NaOH (aq), brine, and anhydrous $Na_2SO_4$. Volatiles were evaporated and the residue was column chromatographed (5 → 10% EtOAc/hexane) to give **C**[11–13] (24.4 mg, 28%) as a light yellow powder: $^1$H NMR (400 MHz, $CDCl_3$) $\delta$ 7.75 (dd, $J$ = 6.0, 3.2 Hz, 1H), 7.81–7.87 (m, 2H), 7.95 (d, $J$ = 8.8 Hz, 1H), 8.25 (d, $J$ = 8.8 Hz, 1H), 8.67 (dd, $J$ = 6.0, 3.2 Hz, 1H); $^{13}$C NMR (101 MHz, $CDCl_3$) $\delta$ 124.41, 125.22, 127.10, 127.28, 127.46, 127.68, 129.00, 130.61, 130.89, 133.29, 134.76, 135.55, 137.75.

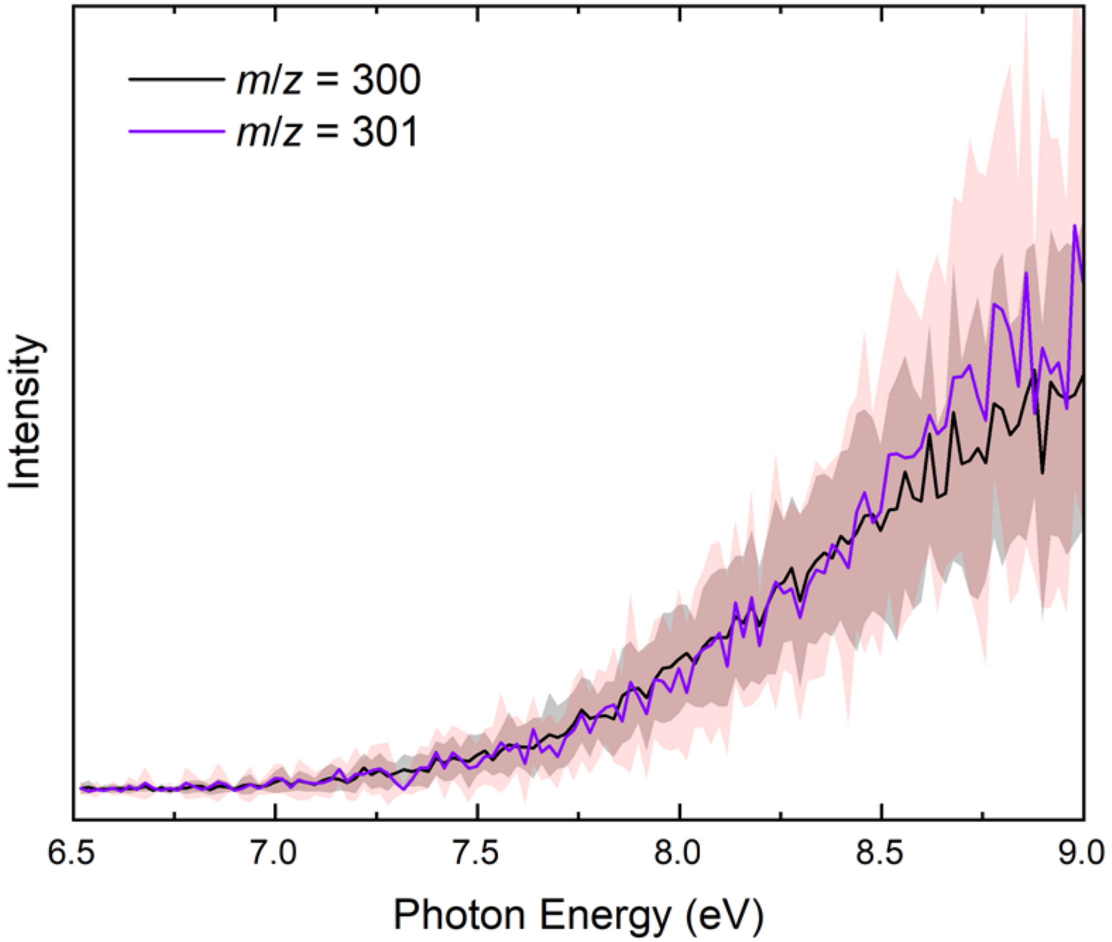


**Supplementary Fig. 2 Photoionization efficiency (PIE) curves for benzocorannulene.** PIE curves relevant to the formation of benzocorannulene at *m*/*z* = 300 ($C_{24}H_{12}$) and *m*/*z* = 301 ($^{13}CC_{23}H_{12}$). Black: experimentally derived PIE curve; purple: $^{13}C$-substituted experimental PIE curve. The overall error bars (*m*/*z* = 300: gray area; *m*/*z* = 301: red area) consist of two parts: 1 σ error of the PIE curve averaged over the individual scans and ±10% based on the accuracy of the photodiode.

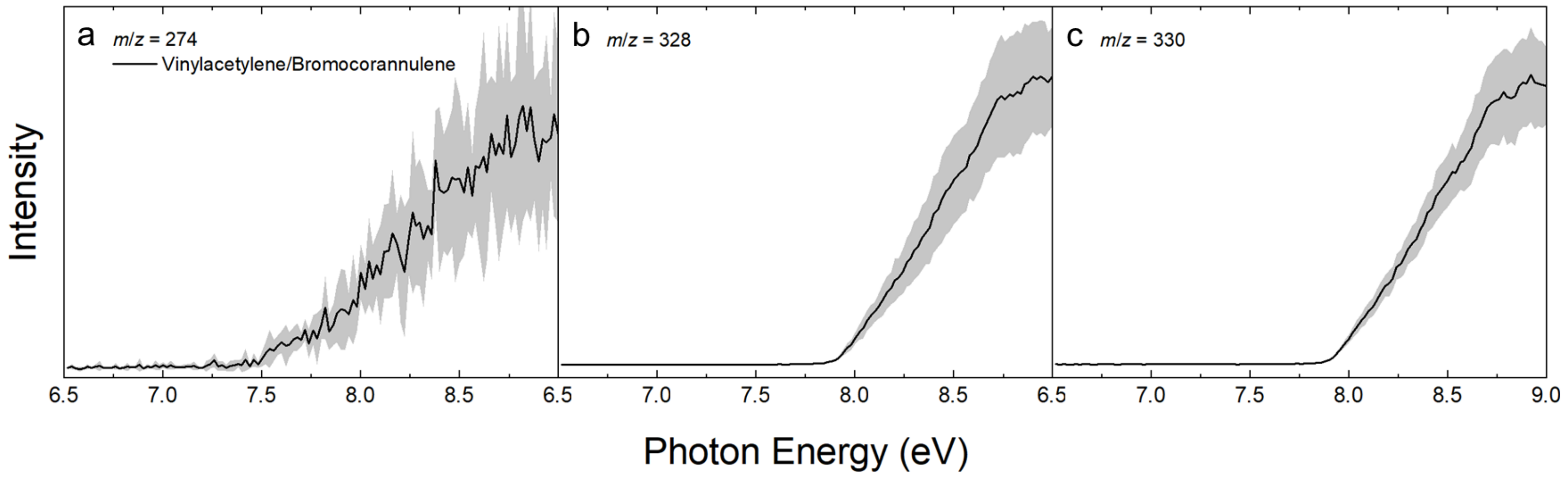


**Supplementary Fig. 3 Photoionization efficiency (PIE) curves for other species.** PIE curves for the vinylacetylene/bromocorannulene system at (**a**) *m*/*z* = 274 ($C_{22}H_{10}$), (**b**) *m*/*z* = 328 ($C_{20}H_9{}^{79}Br$), and (**c**) *m*/*z* = 330 ($C_{20}H_9{}^{81}Br$). The overall error bars (gray area) consist of two parts: 1 σ error of the PIE curve averaged over the individual scans and ±10% based on the accuracy of the photodiode.

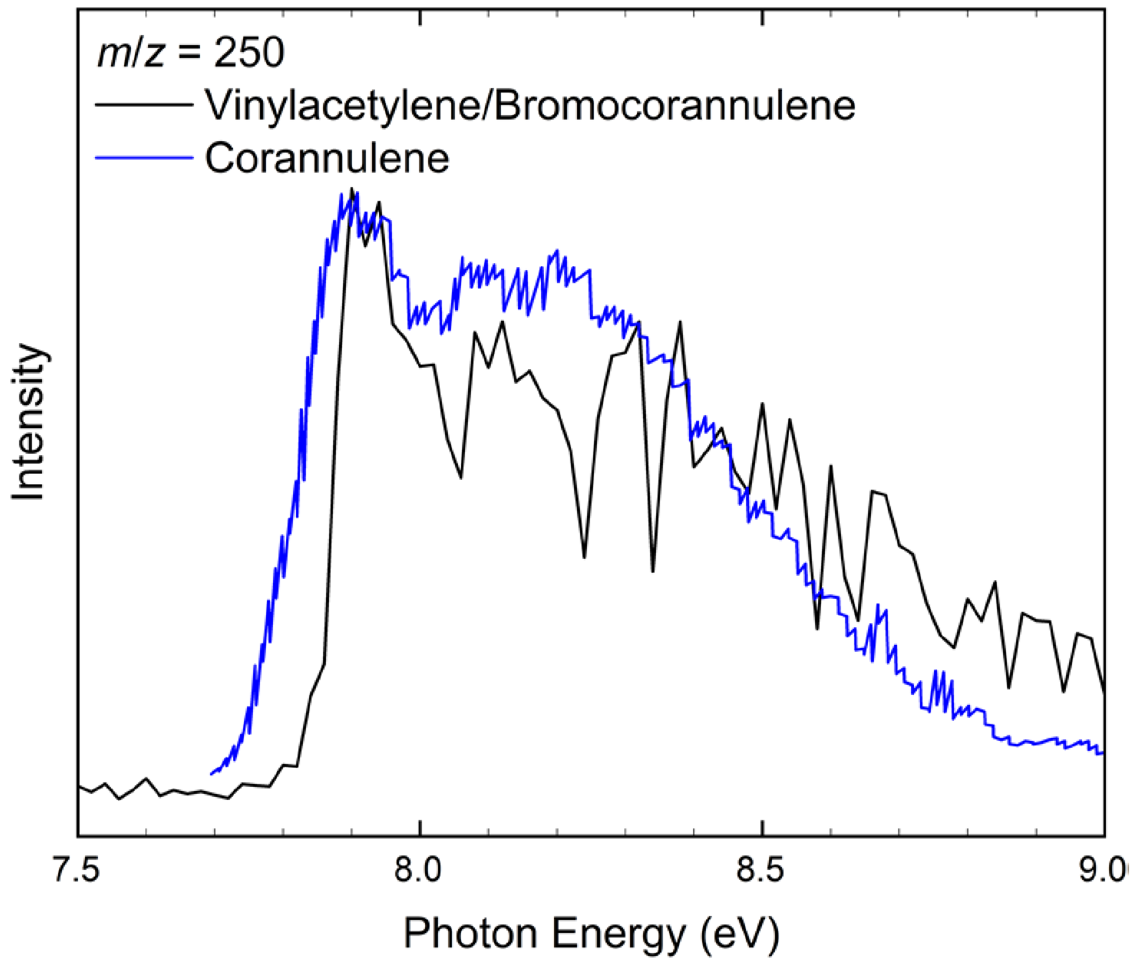


**Supplementary Fig. 4 Mass-selected threshold photoelectron (ms-TPE) spectra for corannulene.** (Black) ms-TPE spectrum for the vinylacetylene/bromocorannulene system at *m*/*z* = 250 ($C_{20}H_{10}$), and (blue) reference photoelectron spectrum of corannulene ($C_{20}H_{10}$) measured by Seiders et al[14].

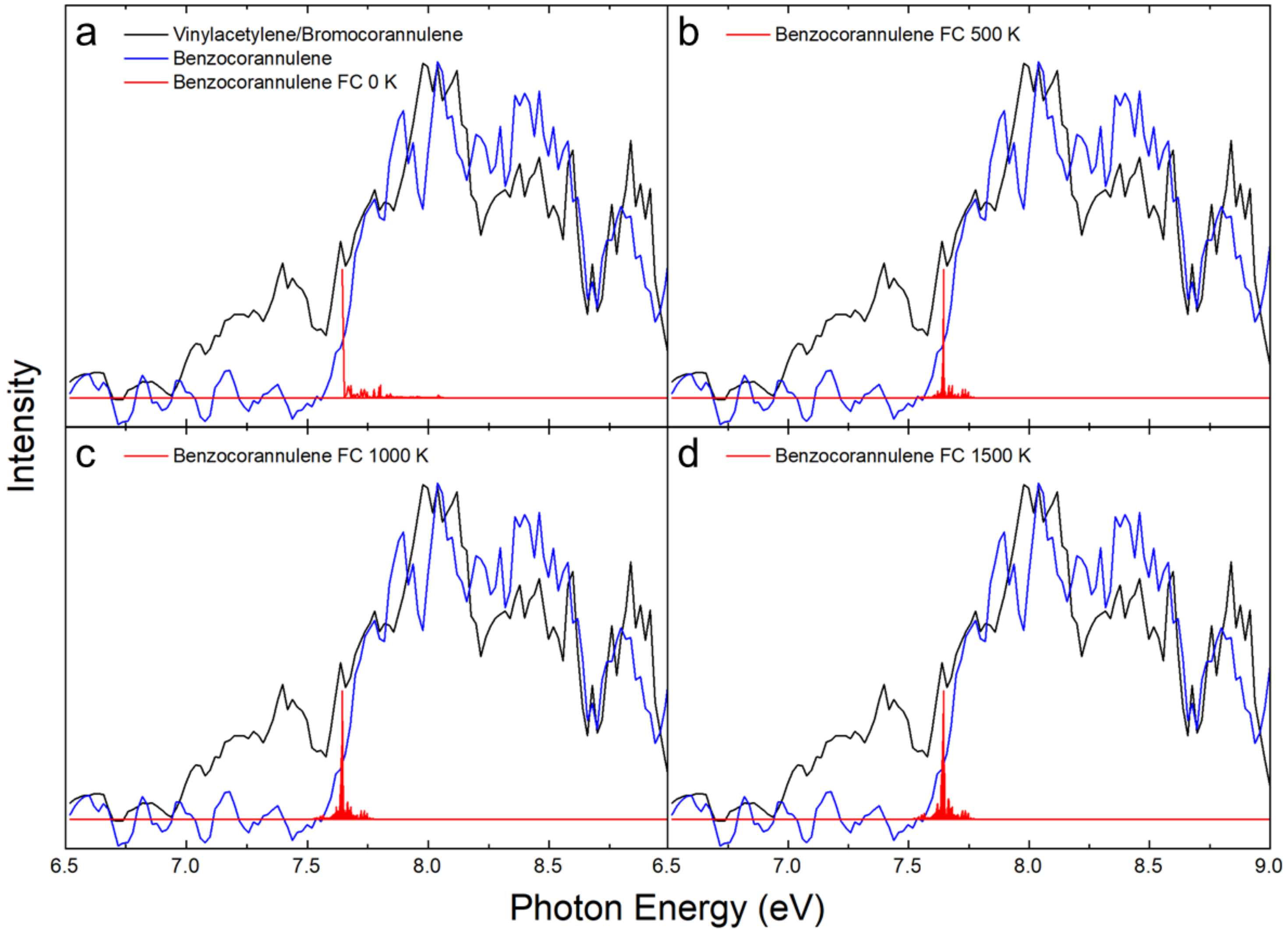


**Supplementary Fig. 5 Mass-selected threshold photoelectron (ms-TPE) spectra for benzocorannulene.** ms-TPE spectra relevant to the formation of benzocorannulene at *m*/*z* = 300 ($C_{24}H_{12}$). Black: experimentally derived ms-TPE spectrum; blue: benzocorannulene ms-TPE spectrum; red: Franck-Condon (FC) simulations for benzocorannulene at (**a**) 0 K, (**b**) 500 K, (**c**) 1000 K, and (**d**) 1500 K.

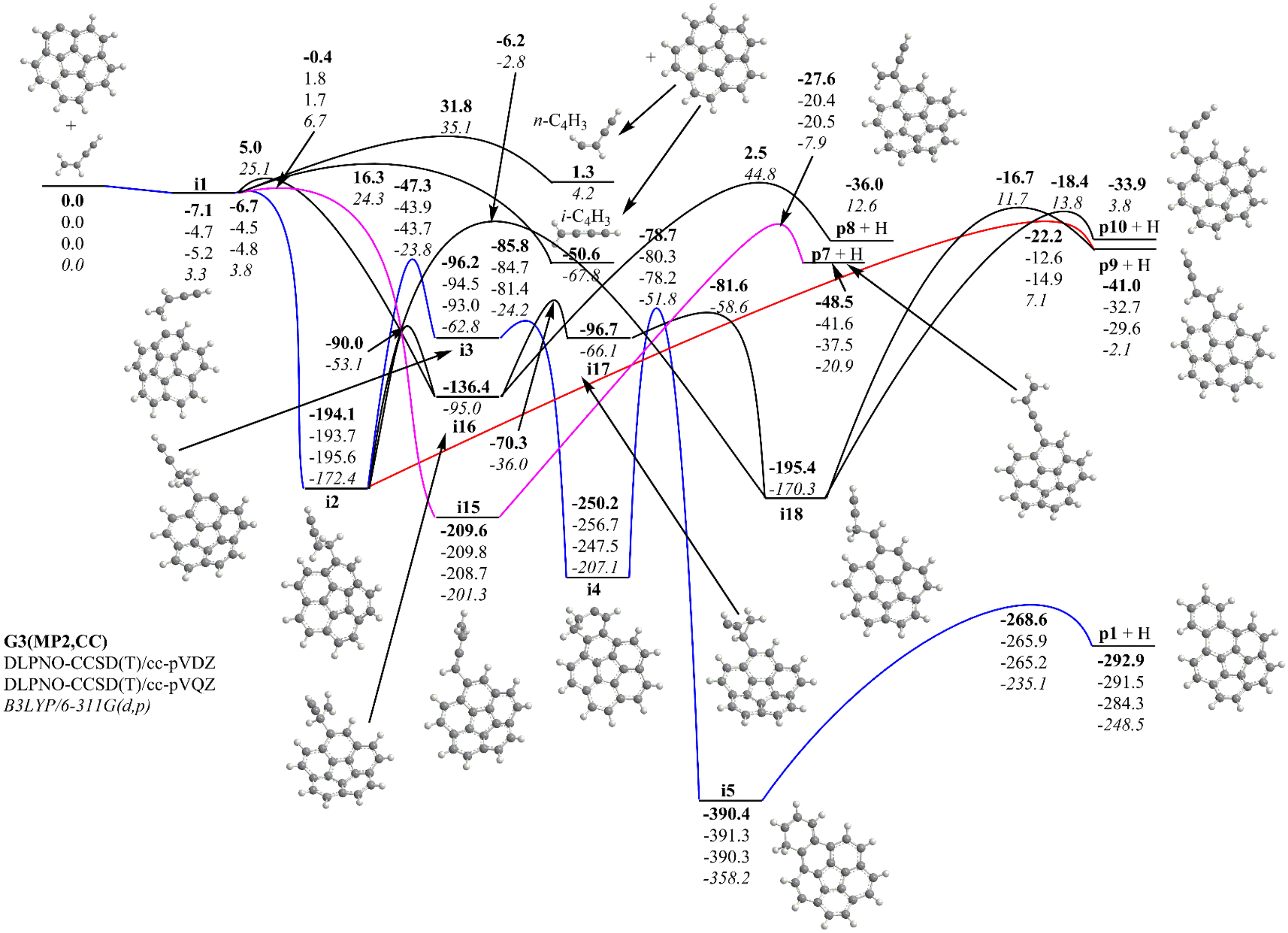


**Supplementary Fig. 6 Full potential energy diagram leading to benzocorrannulene.** Calculated potential energy diagram for the $[C_{20}H_9]^{\bullet}$ plus vinylacetylene reaction. Relative energies of various species are given in kJ mol$^{-1}$ with respect to the initial reactants. Carbon atoms are grey and hydrogen atoms are white.

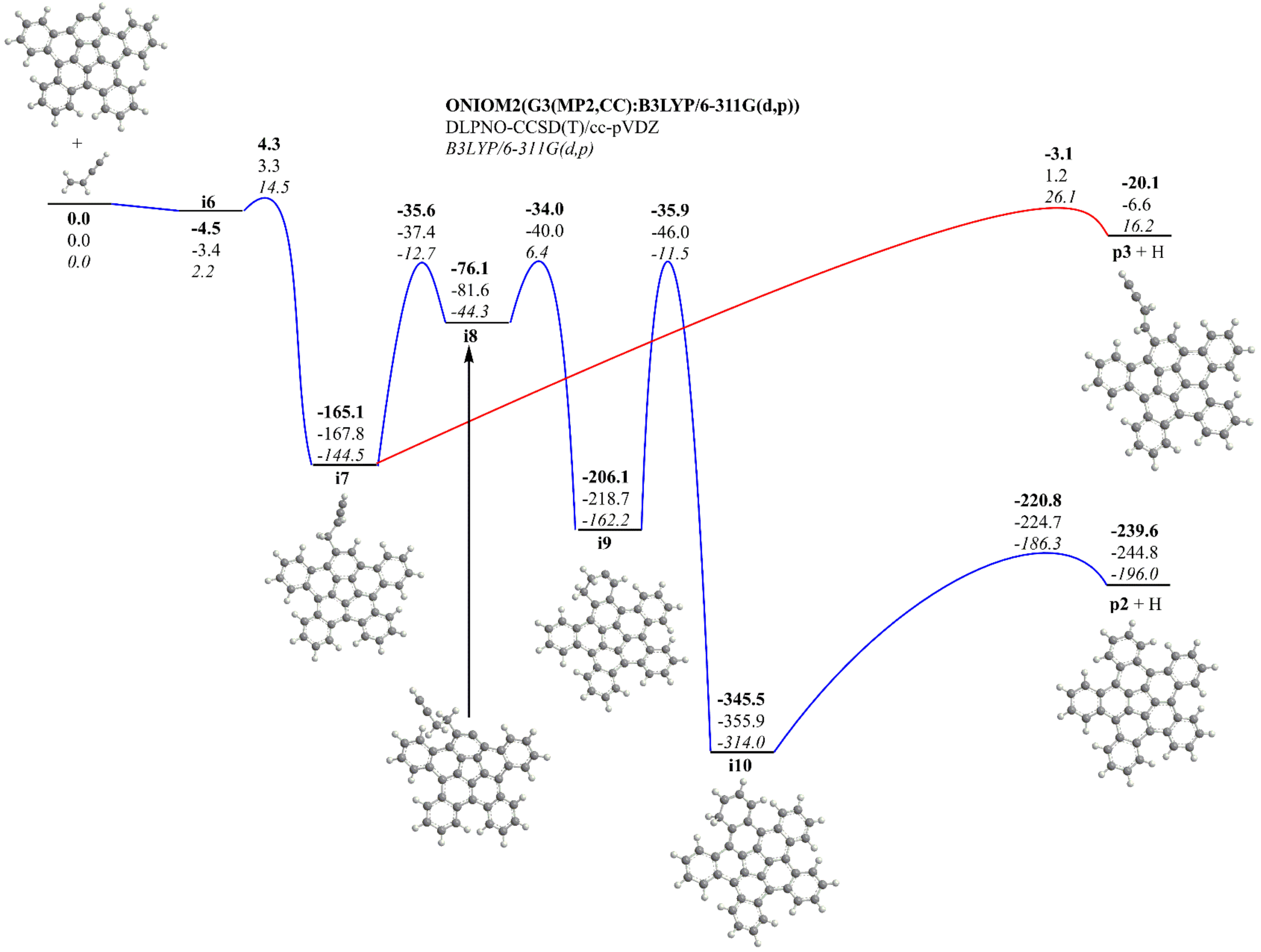


**Supplementary Fig. 7 Full potential energy diagram leading to pentabenzocorannulene.** Calculated potential energy diagram for the most important channels of the $[C_{36}H_{17}]^{\bullet}$ plus vinylacetylene reaction. Relative energies of various species are given in kJ mol$^{-1}$ with respect to the initial reactants. Carbon atoms are grey and hydrogen atoms are white.

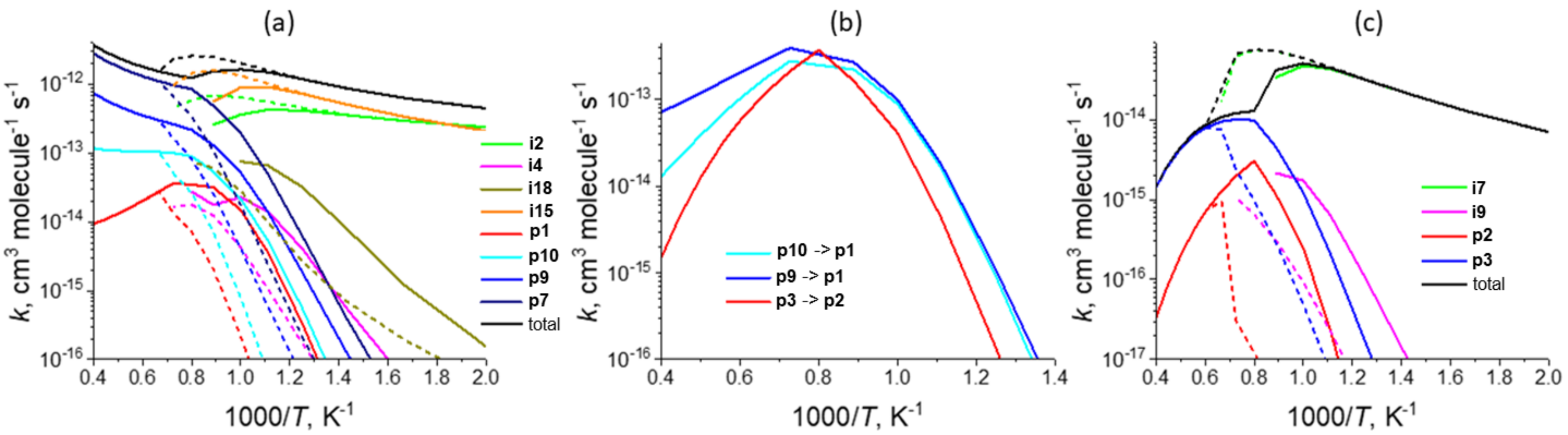


**Supplementary Fig. 8 Reaction rate constants.** Calculated total and individual channel rate constants for the $[C_{20}H_9]^•$ plus $C_4H_4$ reaction (a), for reverse reactions of hydrogen assisted isomerization of the **p10** and **p9** products to **p1** and of **p3** to pentabenzocorannulene **p2** (b), and for the $[C_{36}H_{17}]^•$ plus $C_4H_4$ reaction (c). Solid and dashed lines show rate constants computed at 0.03 and 1 atm, respectively.

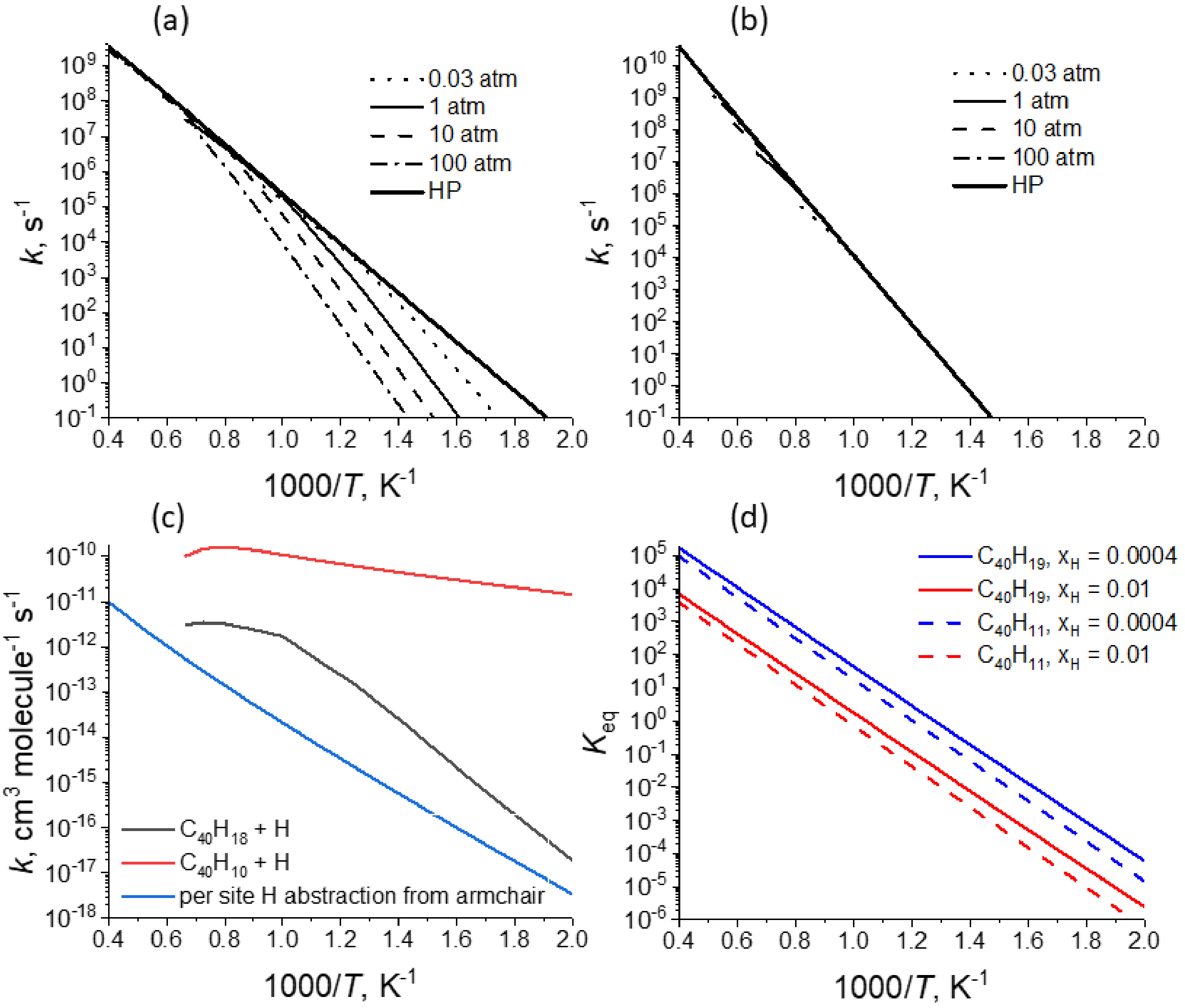


**Supplementary Fig. 9 Unimolecular decomposition rate constants.** Calculated rate constants for unimolecular decomposition of (a) **i11**, (b) **i14**, (c) the reverse $C_{40}H_{18}$/$C_{40}H_{10}$ + H addition reactions at 1 atm in comparison with a recommended per site H abstraction rate constant from a PAH armchair edge[3] (d) equilibrium constants $[C_{40}H_{19}]^{\bullet} \leftrightarrows C_{40}H_{18}$ + H and $[C_{40}H_{11}]^{\bullet} \leftrightarrows C_{40}H_{10}$ + H computed at various mole fractions $x_H$ of hydrogen atoms.

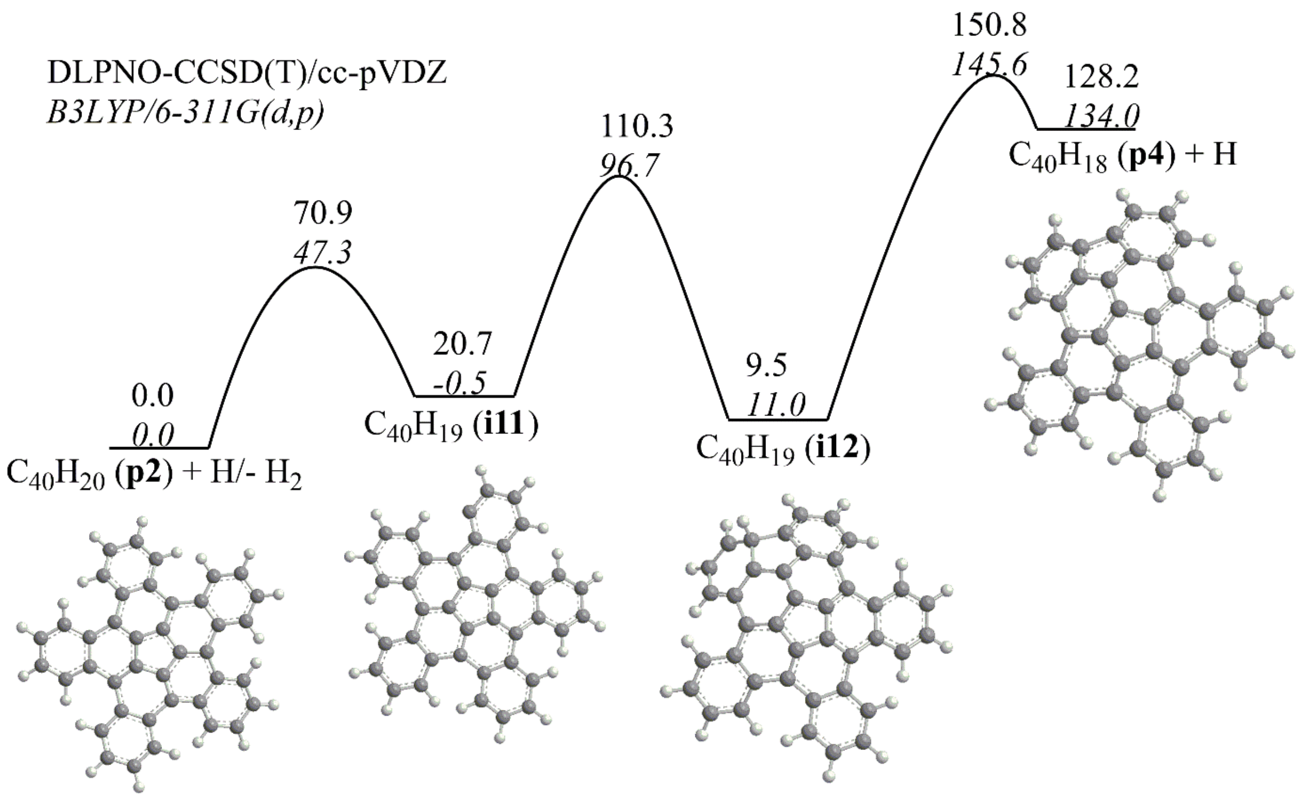


**Supplementary Fig. 10a First cyclodehydrogenation step to the C40 nanobowl.** Calculated potential energy diagrams for the first step in the conversion of pentabenzocorannulene ($C_{40}H_{20}$) to the C40 nanobowl ($C_{40}H_{10}$) via a sequence of five hydrogen abstraction – cyclodehydrogenation mechanisms. Relative energies of various species are given in kJ mol$^{-1}$ with respect to the initial reactants for reaction in the sequence. Carbon atoms are grey and hydrogen atoms are white.

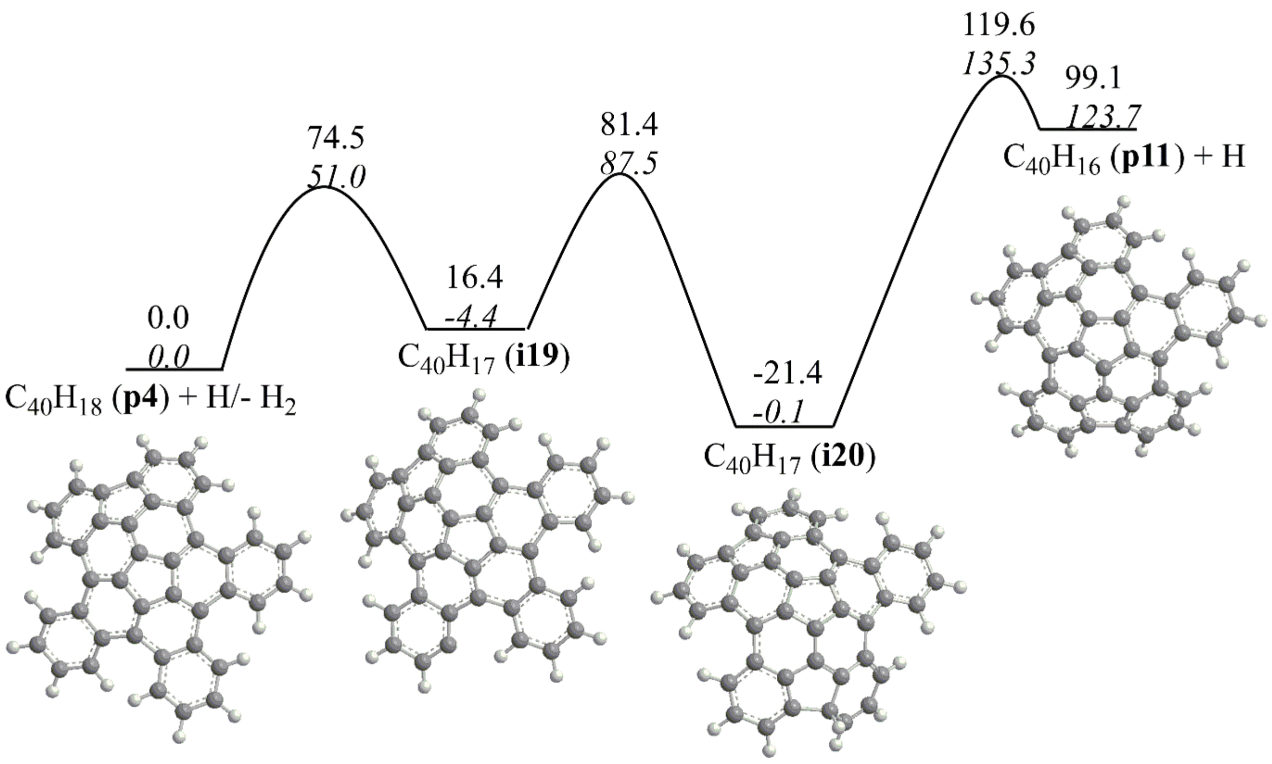


**Supplementary Fig. 10b Second cyclodehydrogenation step to the C40 nanobowl.** Calculated potential energy diagrams for the second step in the conversion of pentabenzocorannulene ($C_{40}H_{20}$) to the C40 nanobowl ($C_{40}H_{10}$) via a sequence of five hydrogen abstraction – cyclodehydrogenation mechanisms. Relative energies of various species are given in kJ mol$^{-1}$ with respect to the initial reactants for reaction in the sequence. Carbon atoms are grey and hydrogen atoms are white.

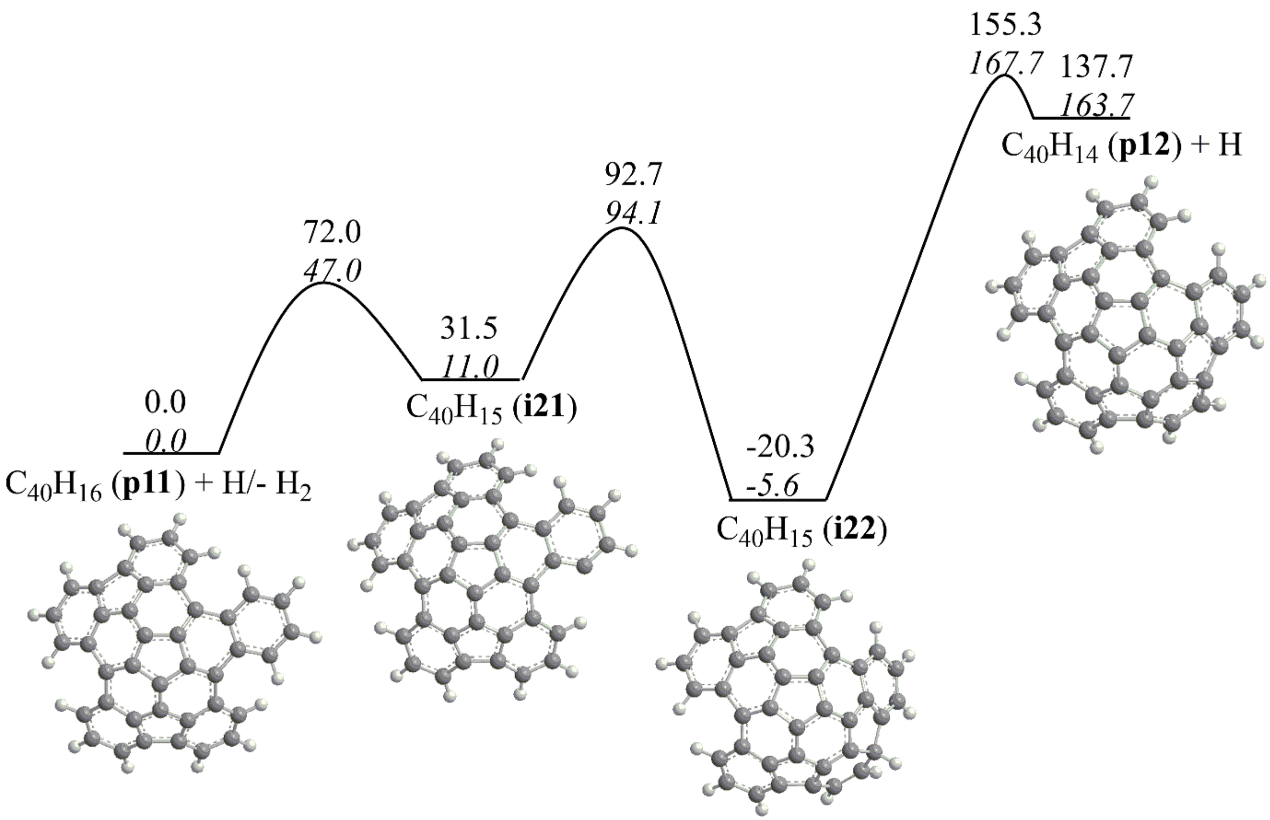


**Supplementary Fig. 10c Third cyclodehydrogenation step to the C40 nanobowl.** Calculated potential energy diagrams for the third step in the conversion of pentabenzocorannulene ($C_{40}H_{20}$) to the C40 nanobowl ($C_{40}H_{10}$) via a sequence of five hydrogen abstraction – cyclodehydrogenation mechanisms. Relative energies of various species are given in kJ $mol^{-1}$ with respect to the initial reactants for reaction in the sequence. Carbon atoms are grey and hydrogen atoms are white.

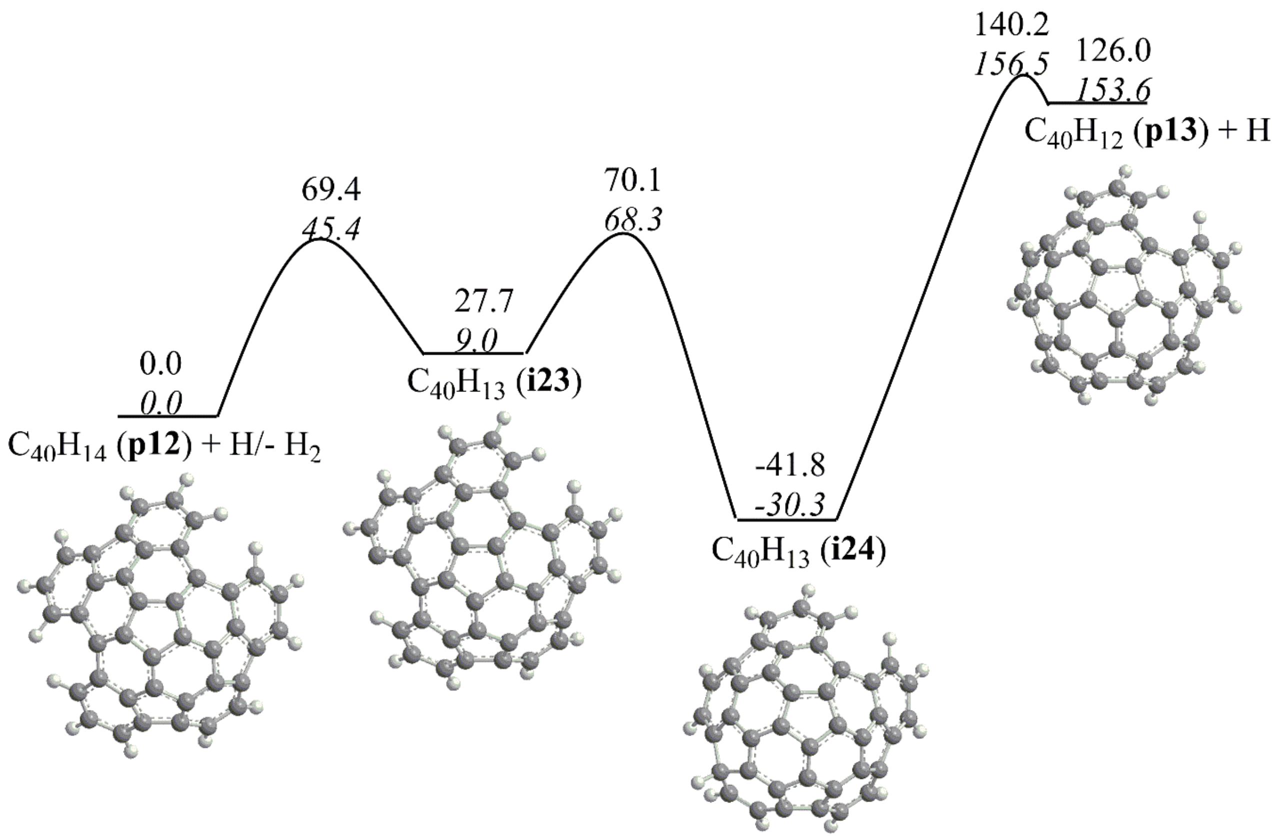


**Supplementary Fig. 10d Fourth cyclodehydrogenation step to the C40 nanobowl.** Calculated potential energy diagrams for the fourth step in the conversion of pentabenzocorannulene ($C_{40}H_{20}$) to the C40 nanobowl ($C_{40}H_{10}$) via a sequence of five hydrogen abstraction – cyclodehydrogenation mechanisms. Relative energies of various species are given in kJ mol$^{-1}$ with respect to the initial reactants for reaction in the sequence. Carbon atoms are grey and hydrogen atoms are white.

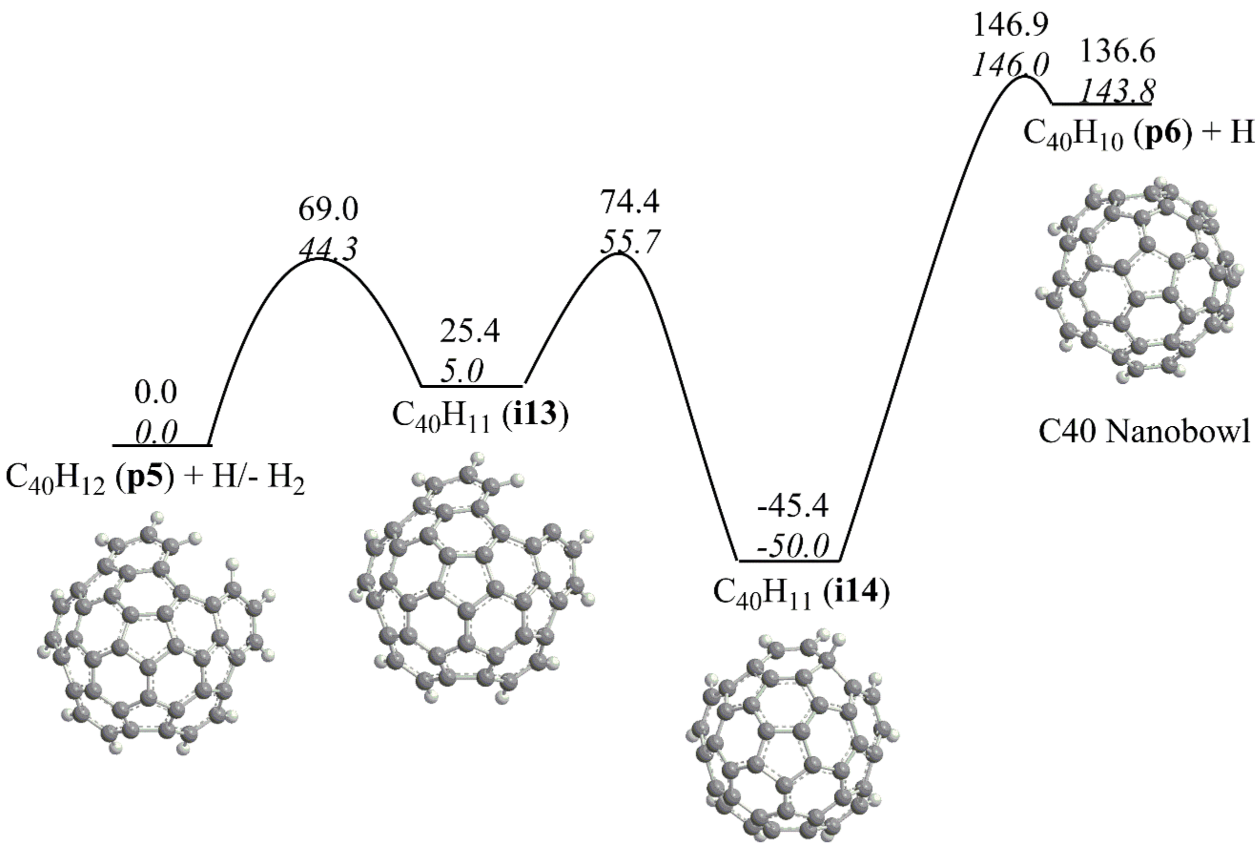


**Supplementary Fig. 10e Fifth cyclodehydrogenation step to the C40 nanobowl.** Calculated potential energy diagrams for the fifth step in the conversion of pentabenzocorannulene ($C_{40}H_{20}$) to the C40 nanobowl ($C_{40}H_{10}$) via a sequence of five hydrogen abstraction – cyclodehydrogenation mechanisms. Relative energies of various species are given in kJ mol$^{-1}$ with respect to the initial reactants for reaction in the sequence. Carbon atoms are grey and hydrogen atoms are white.

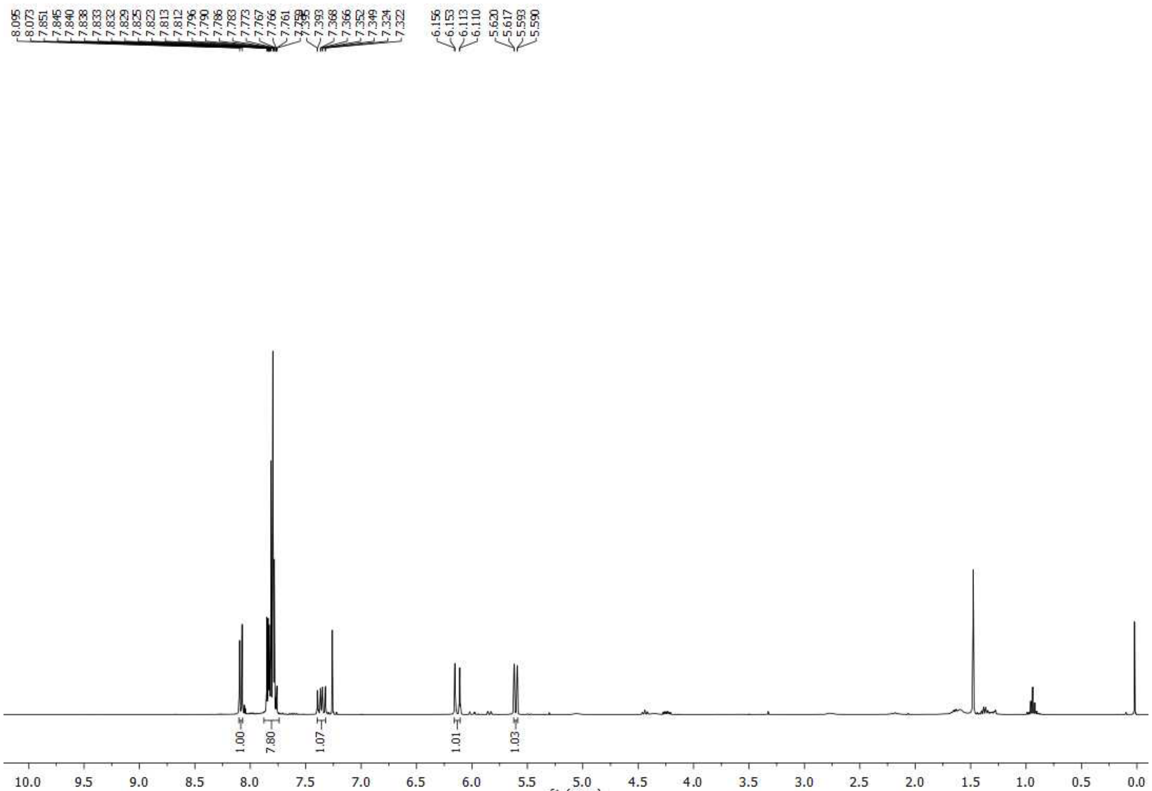

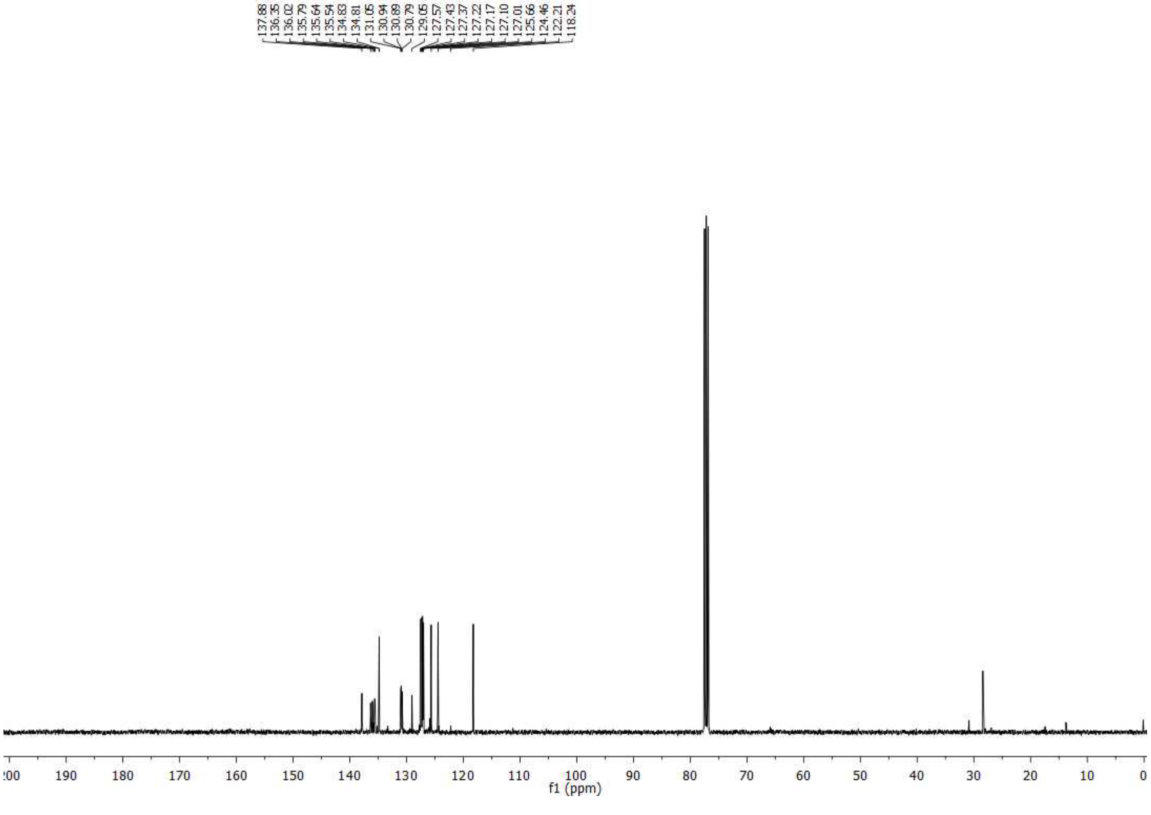

**Supplementary Fig. 11 NMR spectra.** $^{1}H$ NMR and $^{13}C$ NMR spectra of compound **B** in $CDCl_3$.

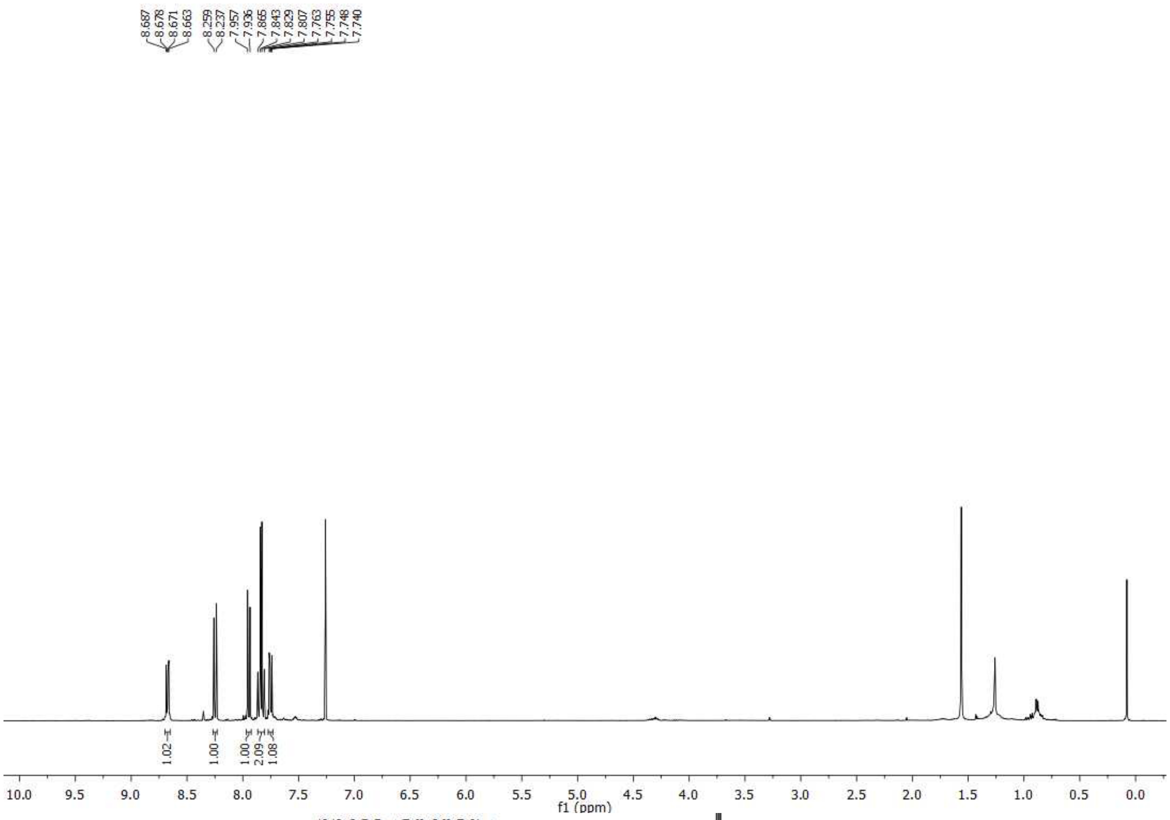

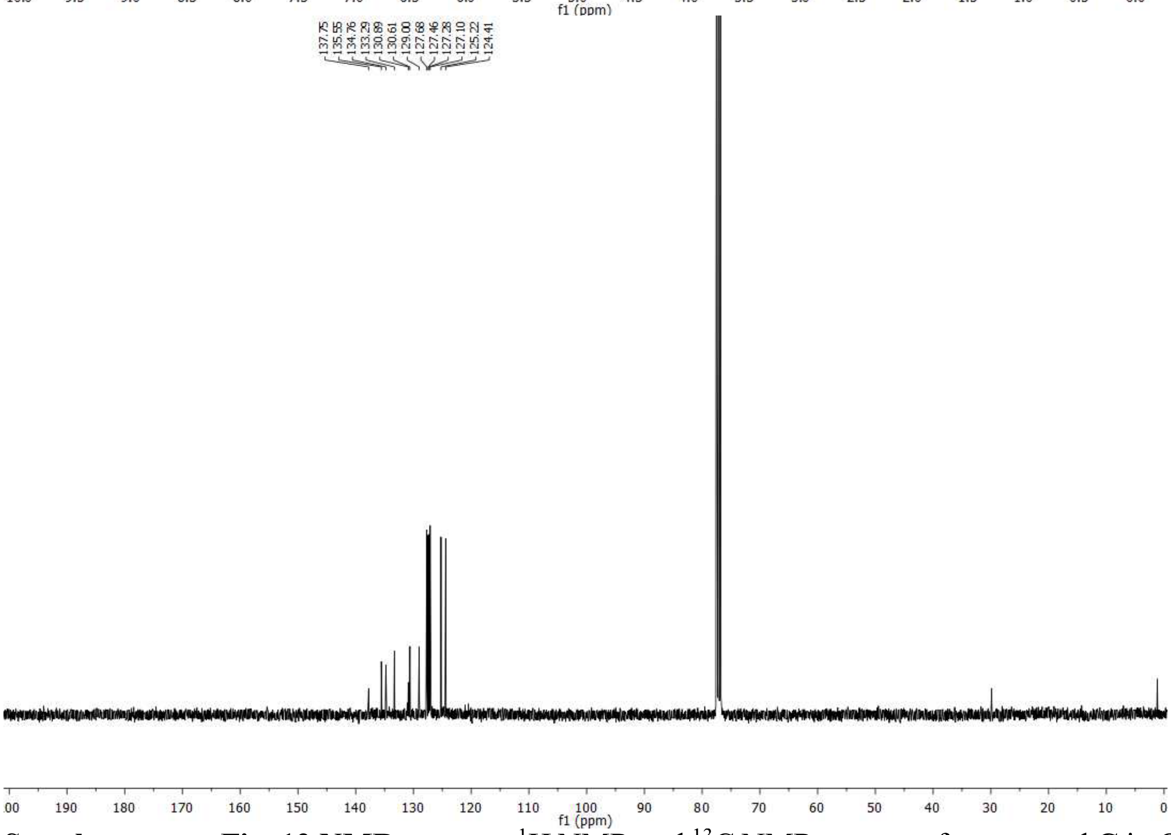

**Supplementary Fig. 12 NMR spectra.** $^{1}H$ NMR and $^{13}C$ NMR spectra of compound **C** in $CDCl_3$.

**Supplementary Table 1.** Relative energies (kJ mol$^{-1}$) of intermediates, transition states, and products in the reactions of phenyl and various PAH radicals with vinylacetylene.

| | phenyl[a] | 1-naphthyl[b] | 2-tetracenyl[c] | 4-phenanthrenyl[d] | corannulenyl[e] | tetrabenzo-corannulenyl[f] |
|---|---|---|---|---|---|---|
| **i1** | -17 | -11 | -9 | -8 | -7.1 (*-4.7*, -5.2) | -4.5 (*-3.4*) |
| **i1-i2** | -0.4 | -2 | -5 | 6 | -6.7 (*-4.5*, -4.8) | 4.3 (*3.3*) |
| **i2** | -187 | -193 | -195 | -162 | -194 (*-194*, -196) | -165 (*-168*) |
| **i2-i3** | -33 | -38 | -39/-42 | -21 | -47 (*-44*, -44) | -36 (*-37*) |
| **i3** | -91 | -91 | -99/-97 | -62 | -96 (*-95*, -93) | -76 (*-82*) |
| **i3-i4** | -58 | -51 | -67/-63 | -26 | -86 (*-85*, -81) | -34 (*-40*) |
| **i4** | -248 | -244 | -261/-250 | -173 | -250 (*-257*, -248) | -206 (*-219*) |
| **i4-i5** | -71 | -56 | -82/-78 | -41 | -79 (*-80*, -78) | -36 (*-46*) |
| **i5** | -375 | -380 | -386/-386 | -352 | -390 (*-391*, -390) | -346 (*-356*) |
| **i5-p1** | -241 | -253 | -229/-264 | -224 | -269 (*-266*, -265) | -221 (*-225*) |
| **p1** + H | -265 | -277 | -243/-285 | -246 | -293 (*-292*, -284) | -240 (*-245*) |
| **i2-p9** | -21 | -16 | -24 | 8 | -22 (*-13*, -15) | -3 (*-1*) |
| **p9** + H | -36 | -29 | -38 | -15 | -41 (*-33*, -30) | -20 (*-7*) |
| **i1-i15** | 5 | 3 | 3 | 9 | -0.4 (*1.8*, 1.7) | |
| **i15** | -208 | -202 | -212 | -179 | -210 (*-210*, -209) | |
| **i15-p7** | -22 | -25 | -27 | -6 | -28 (*-20*, -21) | |
| **p7** + H | -42 | -49 | -46 | -28 | -49 (*-42*, -38) | |

[a]From[1]. The ring annulation product is naphthalene.
[b]From[2]. The ring annulation product is phenanthrene.
[c]From[3]. For the species along the ring annulation pathways, the first number corresponds to the channel leading to the formation of pentacene and the second number corresponds to the channel leading to benzo[a]tetracene.
[d]From[4]. The ring annulation product is [4]-helicene.
[e]The ring annulation product is benzocorannulene. For each species, the first number is calculated at the G3(MP2,CC) level of theory and the numbers in parentheses are obtained at the DLPNO-CCSD(T) level with the cc-pVDZ (italic) and cc-pVQZ basis sets.
[f]The ring annulation product is pentabenzocorannulene. For each species, the first number is calculated at the ONIOM2{G3(MP2,CC):B3LYP/6-311G(d,p)) level of theory and the number in parentheses (italic) is obtained at the DLPNO-CCSD(T)/cc-pVDZ level.

## Supplementary References


1. Zhao, L. et al. A VUV photoionization study on the formation of the simplest polycyclic aromatic hydrocarbon: naphthalene ($C_{10}H_8$). *J. Phys. Chem. Lett.* **9**, 2620-2626 (2018).
2. Zhao, L. et al. Low-temperature formation of polycyclic aromatic hydrocarbons in Titan's atmosphere. *Nat. Astron.* **2**, 973-979 (2018).
3. Zhao, L. et al. A free radical prompted barrierless gas phase synthesis of pentacene. *Angew. Chem., Int. Ed.* **59**, 11334-11338 (2020).
4. Zhao, L. et al. Gas phase synthesis of [4]-helicene. *Nat. Comm.* **10**, 1510 (2019).
5. Semenikhin, A. S. et al. Rate constants for H abstraction from benzo(a)pyrene and chrysene: a theoretical study. *Phys. Chem. Chem. Phys.* **19**, 25401-25413 (2017).
6. Frenklach, M. & Mebel, A. M. On the mechanism of soot nucleation. *Phys. Chem. Chem. Phys.* **22**, 5314-5331 (2020).
7. Frenklach, M., Semenikhin, A. S. & Mebel, A. M. On the mechanism of soot nucleation. III. the fate and facility of the E-bridge. *J. Phys. Chem. A* **125**, 6789-6795 (2021).
8. Xu, B., Guo, T. & Siegel, J. Mono- and *sym*-pentahalogenated corannulenes: reaction optimization & purification by in process product analysis. *React. Chem. Eng.* **7**, 1230-1235 (2022).
9. Mack, J., Vogel, P., Jones, D., Kaval, N. & Sutton, A. The development of corannulene-based blue emitters. *Org. Biomol. Chem.* **5**, 2448-2452 (2007).
10. Muzammil, E. M., Halilovic, D. & Stuparu, M. C. Synthesis of corannulene-based nanographenes. *Comm. Chem.* **2**, 58 (2019).
11. Smith, N. J. Ph.D dissertation, Boston College. Graduate School of Arts and Sciences (2011).
12. Rajeshkumar, V., Lee, Y. T. & Stuparu, M. C. Corannulenecarbaldehyde: high-yielding synthesis by Rieche formylation and facile access to a variety of corannulene derivatives. *Eur. J. Org. Chem.* **2016**, 36-40 (2016).
13. Peng, L. & Scott, L. T. Interconversions of aryl radicals by 1,4-shifts of hydrogen atoms. a synthesis of benzo[a]corannulene. *J. Am. Chem. Soc.* **127**, 16518-16521 (2005).
14. Seiders, T. J., Baldridge, K. K., Siegel, J. S. & Gleiter, R. Ionization of corannulene and 1,6-dimethylcorannulene: photoelectron spectra, electrochemistry, charge transfer bands and ab initio computations. *Tetrahedron Lett.* **41**, 4519-4522 (2000).
