# Supplementary material for: Gas phase synthesis of the C40 nano bowl C40H10
Source: Nat Commun. 2023 Mar 18;14:1527. doi: 10.1038/s41467-023-37058-y (PMC10024697; doi:10.1038/s41467-023-37058-y)
Supplement: Supplementary file 2 — Description of Additional Supplementary files [file 41467_2023_37058_MOESM2_ESM.docx]

**Description of Additional Supplementary Items**

File name: Supplementary Data 1

Description: Input file for RRKM-ME calculations for the [C_20_H_9_]^•^ + C_4_H_4_ reaction using the MESS package.

File name: Supplementary Data 2

Description: Input file for RRKM-ME calculations for the [C_36_H_17_]^•^ + C_4_H_4_ reaction using the MESS package.

File name: Supplementary Data 3

Description: Input file for RRKM-ME calculations for the [C_40_H_19_]^•^ → C_40_H_18_ + H reaction using the MESS package.

File name: Supplementary Data 4

Description: Input file for RRKM-ME calculations for the [C_40_H_11_]^•^ → C_40_H_10_ + H reaction using the MESS package.
